# Spatial expansions and travelling waves of rabies in vampire bats

**DOI:** 10.1098/rspb.2016.0328

**Published:** 2016-06-15

**Authors:** Julio A. Benavides, William Valderrama, Daniel G. Streicker

**Affiliations:** 1 Institute of Biodiversity, Animal Health and Comparative Medicine, University of Glasgow, Glasgow G12 8QQ, UK; 2 Asociación para el Desarrollo y Conservación de los Recursos Naturales, Lima, Peru; 3 Medical Research Council–University of Glasgow Centre for Virus Research, Glasgow G61 1QH, UK

**Keywords:** *Desmodus rotundus*, disease control, zoonoses, spatial dynamics, *Lyssavirus*, landscape heterogeneity

## Abstract

A major obstacle to anticipating the cross-species transmission of zoonotic diseases and developing novel strategies for their control is the scarcity of data informing how these pathogens circulate within natural reservoir populations. Vampire bats are the primary reservoir of rabies in Latin America, where the disease remains among the most important viral zoonoses affecting humans and livestock. Unpredictable spatiotemporal dynamics of rabies within bat populations have precluded anticipation of outbreaks and undermined widespread bat culling programs. By analysing 1146 vampire bat-transmitted rabies (VBR) outbreaks in livestock across 12 years in Peru, we demonstrate that viral expansions into historically uninfected zones have doubled the recent burden of VBR. Viral expansions are geographically widespread, but severely constrained by high elevation peaks in the Andes mountains. Within Andean valleys, invasions form wavefronts that are advancing towards large, unvaccinated livestock populations that are heavily bitten by bats, which together will fuel high transmission and mortality. Using spatial models, we forecast the pathways of ongoing VBR epizootics across heterogeneous landscapes. These results directly inform vaccination strategies to mitigate impending viral emergence, reveal VBR as an emerging rather than an enzootic disease and create opportunities to test novel interventions to manage viruses in bat reservoirs.

## 1. Introduction

Bats are associated with numerous zoonotic pathogens, including SARS Coronavirus and Ebola virus, that have emerged unpredictably into human and domestic animal populations with serious health and economic consequences [1]. A central impediment to forecasting the cross-species transmission of bat viruses and developing strategies for their control is the scarcity of data that could inform how these viruses are maintained in their bat reservoirs [2]. In particular, data from bats are usually collected from small geographical areas over short timescales, whereas the high mobility of bats can enable long-term viral persistence across large spatial scales [3]. By contrast, cross-species infections (‘spillovers’) can span large areas and timescales, but rarely occur at sufficiently high frequency to enable robust inference into transmission dynamics within bats and are often complicated by the possibility of onward transmission within spillover host species [2].

In Latin America, common vampire bats (*Desmodus rotundus*) are the most important source of human and animal rabies, a lethal encephalitis transmitted by the bites of infected animals [4]. The widespread distribution of the vampire bat, from Mexico to Argentina, coupled with a mechanism for cross-species transmission (obligatory blood feeding behaviour) creates high economic, social and public health burdens of rabies across the region [5]. Although post-exposure vaccination of humans and pre-exposure vaccination of livestock are available and efficacious, they are not practiced at large scales [5,6]. Instead, control of vampire bat-transmitted rabies (VBR) uses bat population control, either by indiscriminate killing and roost destruction or by government-led campaigns that use a poisonous paste, ‘vampiricide’, to kill bats that groom treated conspecifics or feed from the wounds of treated livestock [7,8]. The efficacy of these programmes has been questioned by field studies showing that rates of VBR exposure in bats were equivalent or higher in culled colonies and independent of natural variation in bat colony size [9]. Further modelling work showed that apparently counterproductive effects of culling could arise because VBR persists not locally within a bat colony, but by viral dispersal between colonies [10].

Identifying the precise nature of spatial dynamics and how these contribute to long-term viral persistence is critical to improving rabies control. Detection of epizootic expansions or seasonally varying transmission could help anticipate when and where spillover could occur, thereby enabling preventative vaccination of humans and livestock or interventions within the bat population ahead of outbreaks [11,12]. Because rabies is not transmitted among livestock following infection by bats, livestock provide an ideal sentinel to study the spatiotemporal dynamics within the natural bat reservoir. To date, phylogenetic and time-series analyses of VBR in livestock have revealed contrasting patterns. Epizootics were reported in the north of Argentina in the 1970s [13], but more recent analyses show continuous infection of livestock, indicative of enzootic persistence [5,9,12,14–16]. However, incidence varies within enzootic zones and is speculated to be affected by natural and anthropogenic processes. On the one hand, livestock vaccination or bat culls could conceivably decrease the burden and transmission of VBR, respectively, if practiced intensively. Yet, land use and climate changes could offset these gains by providing greater availability of livestock (a key food resource for bats) and increasing minimal winter temperatures, which together limit the size and geographical range of vampire bats [17]. If VBR outbreaks are increasing, determining whether increases are caused by spatial expansions into previously uninfected areas or intensified transmission within enzootic zones is critical to appropriately allocate vaccines, surveillance and educational campaigns.

The national surveillance system of Peru has documented the time and place of rabies infections in largely unvaccinated livestock populations since 2004. Because rabies transmitted by dogs is restricted to a small, isolated region of Peru, virtually all livestock cases are spillover infections from vampire bats [18,19]. This unique dataset creates a remarkable opportunity to elucidate the spatiotemporal dynamics underlying the perpetuation of VBR in bat populations and thereby inform control strategies across Latin America. Here, using high-resolution time-series data on VBR outbreaks, we: (i) resolve the spatiotemporal patterns underlying previously undetected increases in the burden of VBR in Peru, (ii) test whether increases are caused by spatial expansions or intensified transmission within historically enzootic zones, and (iii) assess patterns of how spatial expansions occurred across the landscape. Next, in an area of intensive VBR transmission in the Andes, we pair time-series data with field data on bat–livestock contacts, vaccination and reporting practices to (iv) estimate the velocity of advancing epizootics of VBR on a heterogeneous landscape, and (v) forecast impending spillover risks to humans and livestock.

## 2. Material and methods

### (a) Datasets

#### (i) National reports of rabies infections in livestock from 2003 to 2014

Suspected outbreaks of rabies were reported by farmers to one of the 103 satellite offices of National Service of Agrarian Health of Peru (SENASA) for sample collection and laboratory testing for rabies infection (*n* = 2269, see Data accessibility section). Because we focused on the spatiotemporal dynamics of VBR rather than the intensity of outbreaks, we analysed data as outbreaks (defined as one or more laboratory-confirmed rabies infection on a farm) rather than as individual cases; however, most outbreaks (83%) involved only a single sick or dead animal.

#### (ii) Spatial distribution of bat bites on livestock

In the southern Andes, we used reports of bat bites on livestock (a proxy for vampire bat presence), combined with national census data, to assess the patchiness of bats on the landscape. The spatial distribution of bat bites on livestock was collected by the Regional Government of Apurimac by conducting visual inspection of livestock in 169 rabies enzootic communities (1–4 farms per community) within two valleys (Chalhuanca and Rio Apurimac) from June 2014 until July 2015 [20]. We hypothesized that patchy bat distributions would require longer bat flights that could lead to accelerated or punctuated spatial spread of VBR, whereas the continuous presence of bats might enable slower, gradual spread.

#### (iii) Questionnaire data from vampire bat-transmitted rabies-free communities

We used oral questionnaires to evaluate the presence of vampire bats feeding on livestock, knowledge of rabies and vaccination practices in communities that did not report outbreaks in the national surveillance dataset. We interviewed 60 farmers in six districts in the Chalhuanca valley and 30 farmers in two districts in the Rio Apurimac valley using a standardized questionnaire (see the electronic supplementary material for details and ethical approval information).

### (b) Spatiotemporal patterns in the burden and distribution of vampire bat-transmitted rabies

Spatial dynamics of VBR were explored by calculating the number of outbreaks per region from 2003 to 2014, the annual number of districts infected, the cumulative number of districts that reported rabies for the first time each year and the percentage of districts that had a neighbouring district reporting rabies prior to its first report. We tested whether the average elevation of outbreaks changed through time in each region of Peru using generalized linear models (GLM) with normally distributed errors. The Akaike's information criterion (AIC) was used for model selection (chosen a difference of more than two AIC points), and the significance of each factor within the selected model was tested using a Wald test [21]. We also tested the overall effect of year on outbreak elevation using a generalized linear mixed model (GLMM) with region as a random effect using the glmmADMB package of R. District and elevation maps were obtained from the GADM database (gadm.org) and the CGIAR-CSI (http://www.cgiar-csi.org/) respectively, using the *raster* package of R. Temporal autocorrelation analyses were used to investigate the seasonality of VBR outbreaks using the *acf* function of R, which allows significance testing different time lags of autocorrelation.

### (c) Estimating the velocity of advancing vampire bat-transmitted rabies epizootics

The large number of VBR outbreaks in the neighbouring regions of Apurimac, Ayacucho and Cusco (AAC, 62% of all outbreaks in Peru) provided a high-resolution dataset to resolve the epidemiological dynamics underlying spatial expansions detected at the national level. Changes in the infected area over time were estimated using annual kernel densities with the *bkde2D* function of the *KernSmooth* package of R (bandwidth = 0.15 and grid = 200 × 200 km) [22]. In two valleys where data suggested an epizootic advance (Chalhuanca and Rio Apurimac, figure 4*b*), we tested whether spatial expansions followed a wave-like pattern using linear regressions between the time and the distance of outbreaks from the predicted index case.

Because VBR persisted locally after invading new areas, we divided the landscape into equidistant hexagonal bins using the *hexbin* function (xbins set to 6) of the *hexbin* package in R and analysed the month of the first outbreak in each cell. We incorporated effects of spatial heterogeneity on VBR spread by calculating distances to index cases as least-cost distances based on a landscape resistance model, defined as the minimum distance along elevations <3600 m, the maximum recorded elevation of *D. rotundus* roosts in the area (see the electronic supplementary material). A sensitivity analysis for this assumption using 200 randomly selected elevation thresholds from 3500 to 4500 m for each of the two valleys is provided in the electronic supplementary material. The index cases of each travelling wave were estimated by simulating 5000 random hypothetical origins of each wave and choosing the origin that maximized the *R*^2^ of each linear regression [23]. Wavefront speeds were calculated as the inverse of the slope of the linear regression with the intercept set to zero. We evaluated the sensitivity of wavefront speeds to the inferred origin by bootstrap resampling (with replacement) the hexbins used in the main analysis. In each of 200 bootstrap replicates in each valley, we evaluated 5000 random hypothetical origins and identified the origin location that maximized the *R*^2^ of the distance/time regression. We then compared the distributions of bootstrapped wavefront speeds and *R*^2^ to the main analysis.

We used the bat bite distribution data (dataset ii) to evaluate whether the patchiness of bats on the landscape could influence wavefront speeds. We divided each valley into hexagonal cells with a specific 5 km radius (assumed as a conservative distance for bat dispersal) [24]. Within each cell, we calculated the number of towns from the 2014 census, the number of communities visited for bite inspection and whether the community reported at least one bat bite. We tested whether the valleys differed on the number of towns per cell and the percentage of communities reporting bites per cell using a Wilcoxon test.

### (d) Forecasting cross-species transmission from bats to livestock

In the two valleys where we quantified wavefront speeds of VBR, we predicted the spatial spread of future outbreaks from 2015 to 2017. Forecasts used the slope of the linear regression to predict the arrival of rabies to each locality according to the least-cost distance of each locality to the inferred origin. As a within-data validation of our predictions, we developed a training model based on the first 75% of available data and applied this to predict the remaining 25% of rabies arrival dates. Details of the cross-validation analysis are given in the electronic supplementary material. All analyses were performed in R [25].

## 3. Results

### (a) Spatial expansions underlie a recent doubling of rabies spillover to livestock

**Figure 1.**
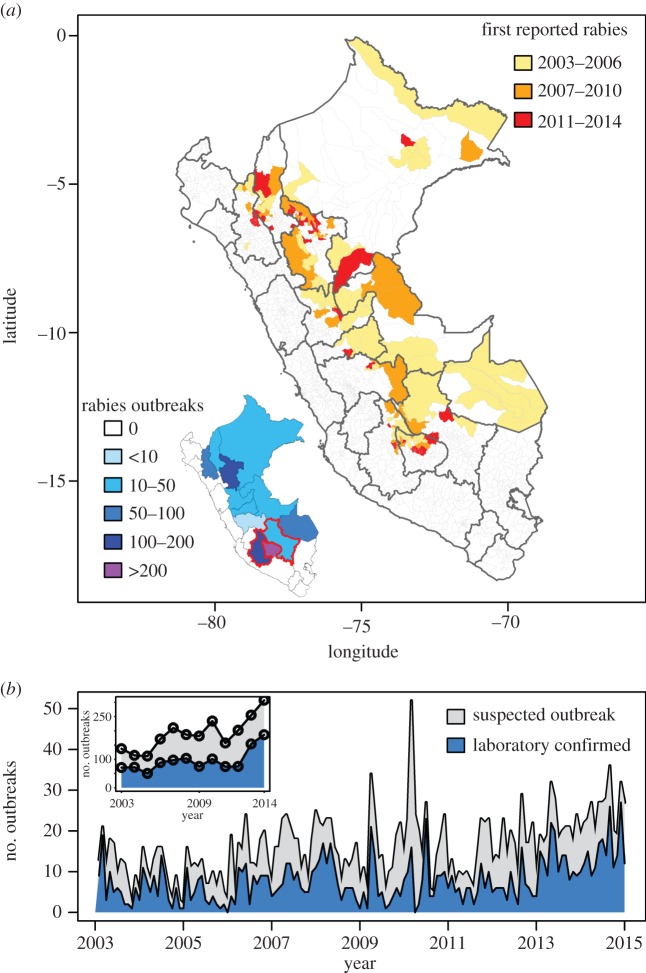
Spatial and temporal patterns of VBR outbreaks in Peru. (*a*) Times of VBR onset at the district level (main map) and the net number of confirmed outbreaks at the regional level (inset). Red lines show the AAC, including the Apurimac, Ayacucho and Cusco regions. (*b*) Pattern of monthly (main figure) and yearly (inset figure) outbreak reports across Peru, excluding regions where dog rabies is enzootic (Puno) and two regions on the coast (Lima and Ancash) that reported small numbers of unconfirmed outbreaks.

In total, 1146 (51%) of 2265 suspected outbreaks between 2003 and 2014 were confirmed rabies positive using the fluorescent antibody test, making VBR the most common notifiable disease in Peruvian livestock (electronic supplementary material, figure S5*a*). Cattle were infected in 91% of the confirmed outbreaks, followed by horses (4%), goats (2%), sheep (1%) and other assorted species (2%, 11 pigs, four buffalos and one camelid). Outbreaks were widespread across the Andes and Amazon, with 14 of 25 governmental regions reporting at least one suspected outbreak in livestock, and 12 regions reporting from 7 to 470 laboratory-confirmed outbreaks (figure 1*a*). Temporal autocorrelation analysis revealed no seasonality in VBR spillover to livestock at either the national or the regional level.

At the national level, the number of VBR outbreaks began a consistent increase in 2011, with a near doubling of outbreaks by 2014 (figure 1*b*). This increase is unlikely to be caused by changes in reporting: the 56 other livestock diseases tracked by the same surveillance system increased from the establishment of the system in 2003 until 2008, but remained stable in later years when rabies reports intensified (electronic supplementary material, figure S5*b*). Furthermore, in a linear model including the effects of the number of other diseases reported and year on the annual number of rabies outbreaks, year (*F*_1,9_ = 11.1, *p* < 0.01), but not outbreaks of other diseases (*F*_1,9_ = 0.64, *p* > 0.1), was significant (full model *R*^2^ = 0.7). Increases in VBR outbreaks were not geographically ubiquitous. Outbreaks declined or were stable in the Amazon, but became more frequent in the Andes, particularly in three regions characterized by valleys with high agricultural production surrounded by high elevation peaks: Apurimac (470 outbreaks, 41% of the national total), Ayacucho (197, 17%) and San Martin (128, 11%) (figure 1*a*; electronic supplementary material, figure S6).

**Figure 2.**
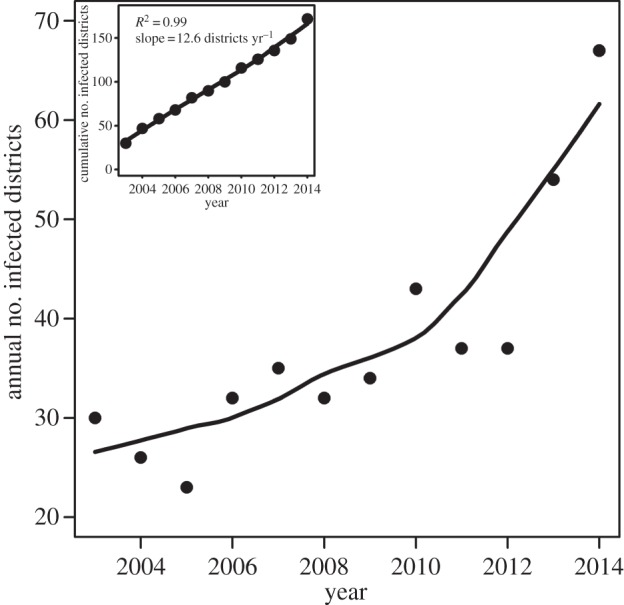
National spatial expansion of VBR. Annual (main figure) and cumulative (inset figure) number of governmental districts reporting VBR outbreaks from 2003 until 2014. Lines show values predicted by the loess function in R.

The national increase in rabies outbreaks occurred concurrently with spatial expansions into historically uninfected areas. The cumulative number of districts with confirmed cases more than tripled between 2003 and 2014, such that on average VBR spread into 12 (standard deviation, s.d. = 0.319) previously uninfected districts per year (figure 2, inset), a pattern also observed at the monthly level (1 district month^−1^, s.d. = 0.007). The number of infected districts also increased in annual (non-cumulative) reports, with most dramatic spatial expansions coinciding with the increase in outbreaks following 2011 (figures 1*b*,2). Hotspots of spatial expansion occurred in the regions of San Martin, Amazonas and Cajamarca in the north of Peru (5.2 districts yr^−1^, s.d. = 0.36) and in Apurimac, Ayacucho and Cusco in the south (4.1 districts yr^−1^, s.d. = 0.12). By contrast, few new districts were infected in the Amazonian regions of Loreto, Ucayali and Madre de Dios (1.4 districts yr^−1^, s.d. = 0.21), where no new districts were infected in the last 3 years. Consistent with epizootic spatial expansions from enzootic zones, 85% of newly infected districts had a neighbouring district infected in the same or previous year.

**Figure 3.**
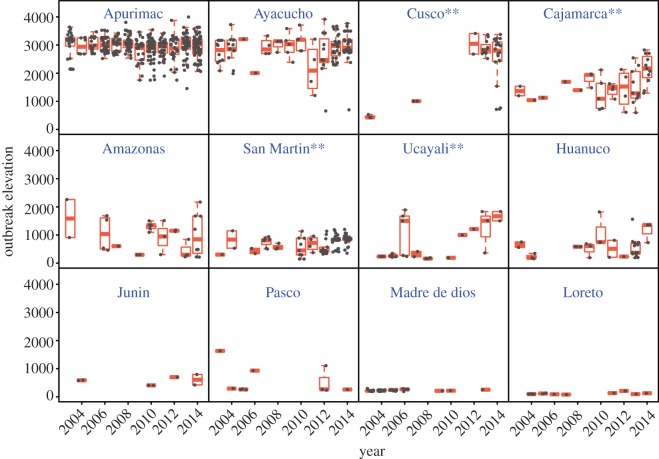
Changes in the geographical elevation of rabies outbreaks in each region. Each panel shows the annual distribution of the elevation of observed outbreaks in each region between 2003 and 2014. Regions are sorted by decreasing order of average elevation. Boxplots show median, second and third quartiles, whereas grey dots present the raw data. Asterisks highlight regions where increases in outbreak elevation were significant in our GLM (*p* < 0.05). (Online version in colour.)

Significant increases in the elevation of VBR outbreaks occurred in regions containing transitions from the Amazon rainforest into the Andes mountains (i.e. Cusco, Cajamarca, San Martin and Ucayali). By contrast, the mean elevation of rabies outbreaks was unchanged in regions with only high (i.e. Apurimac and Ayacucho in the Andes) and low (i.e. Madre de Dios and Loreto in the Amazon) elevations (figure 3). The model with a region by year interaction was more strongly supported than year + region (ΔAIC = 91.2), year alone (ΔAIC = 1790.33) and region alone (ΔAIC = 96.05). Our GLMM showed that, controlling for region as a random effect, the elevation of outbreaks generally increased through time (*z*-value = 2.93, *p* < 0.01). However in several regions (e.g. Ayacucho, Cusco), the minimum elevation of outbreaks decreased through time concurrently with increases in the maximum elevation of outbreaks, creating a funnel pattern which indicates that downhill invasions also occurred (figure 3).

### (b) Velocities of travelling waves of vampire bat-transmitted rabies in heterogeneous landscapes

**Figure 4.**
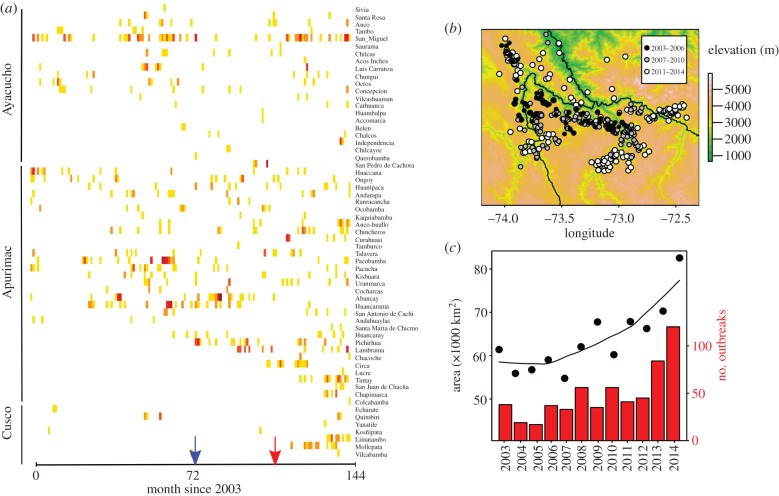
Spatial expansions and increasing burden of VBR in Andean valleys of Apurimac, Ayacucho and Cusco (AAC). (*a*) Monthly VBR outbreaks from 2003 to 2014 in 61 infected districts in AAC. Each row represents the number of outbreaks in an individual district through time. Districts were sorted according to the latitude of their centroid (with the most northern district at the top of the panel) within each region. Darker reds indicate larger numbers of cases (maximum = 8), white indicates the absence of VBR detections. Arrows illustrate the beginning of the wave-like spread for the Chalhuanca (blue) and Rio Apurimac (red) valleys shown in figure 5. (*b*) Detailed spatial locations of outbreaks from 2003 to 2014. (*c*) Number of outbreaks per year and estimated area covered according to kernel density estimates.

In the AAC, kernel density estimates showed a 74% increase in the spatial extent of VBR between 2003 and 2014, equivalent to a newly infected area of approximately 21 000 km^2^ (figure 4*c*). Two different spatiotemporal dynamics of VBR were observed. In the north of the AAC (e.g. San Miguel, Huanipaca, Huancarama districts), VBR persisted enzootically via sporadic outbreaks through the duration of the time series, consistent with metapopulation dynamics proposed by Blackwood *et al*. [10] (figure 4*a,b*). By contrast, VBR only recently invaded districts in the south or southwest (e.g. Tintay, Mollepata, Circa).

**Figure 5.**
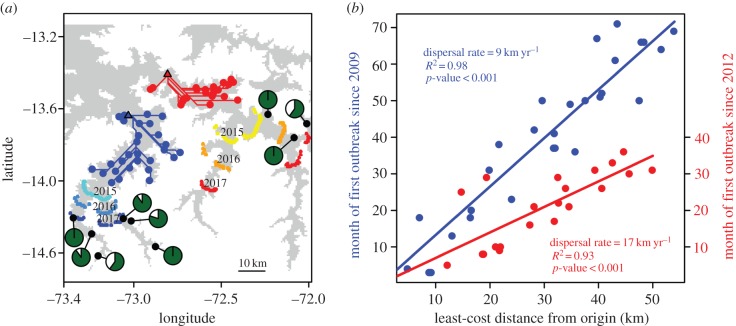
Wave-like spread and outbreak forecasting in two valleys of Peru. (*a*) Maps of Chalhuanca (left) and Rio Apurimac (right) valleys showing the least-cost paths (blue and red lines) from the centre of each cell where an outbreak occurred to the predicted origin of each wave (triangles) and the predicted limits of outbreaks for the end of 2015, 2016 and 2017, respectively. Predicted locations (blue and red colour palettes) were drawn by simulating random coordinates that matched the predicted least-cost distance from linear models. Grey shading indicates altitudes below 3600 m, where vampire bats occur. Pie charts show the percentage (green) of farms where vampire bat bites occurred on livestock for each of nine communities (10 questionnaires per community). (*b*) Linear regressions between the month of the first confirmed VBR outbreak in each cell and the least-cost distance from the inferred outbreak origins in the Chalhuanca (left, blue points) and Rio Apurimac (right, red points) valleys.

In the valleys of Chalhuanca and Rio Apurimac, strong linear relationships between the temporal and the least-cost spatial distance from each outbreak to the predicted index cases revealed wave-like expansions of VBR (figure 5). By the end of 2014, these waves had travelled 39 km in Chalhuanca (94 outbreaks since 2009) and 54 km in Rio Apurimac (45 outbreaks since 2012). Dispersal rates were strikingly consistent through time, as evidenced by high *R*^2^ values in linear models within each valley (*R*^2^ = 0.98 and 0.93 for Chalhuanca and Rio Apurimac, respectively; figure 5*b*). Interestingly, VBR is spreading through bat populations nearly twice as rapidly in the Rio Apurimac valley as in Chalhuanca (17.2 km yr^−1^ (95% confidence interval (CI): 15.3–19.7) versus 9.1 km yr^−1^ (95% CI: 8.6–9.7); figure 5*a*). Uncertainty in the origin locations had little influence on wavefront speeds. Bootstrap estimates revealed similar speeds across all plausible origin locations, with estimations ranging from 14.3 to 20.4 km yr^−1^ (*R*^2^: 0.87–0.98) in Rio Apurimac and from 6.7 to 11.2 (*R*^2^: 0.96–0.99) in Chalhuanca (electronic supplementary material, figure S1). Likewise, estimates of wavefront speeds were insensitive to elevation thresholds in the landscape resistance models, with values across thresholds in the range of 16.5–17.9 km yr^−1^ (*R*^2^: 0.92–0.93) in Rio Apurimac and 7.8–9.2 (*R*^2^: 0.97–0.98) in Chalhuanca (electronic supplementary material, figure S2).

The consistency of wavefront speeds within each valley suggests that VBR spreads predominately by short distance dispersal through a continuously occupied landscape, rather than by punctuated spread driven by long distance flights of infected bats. Census data showed no significant difference between valleys in the number of towns per 5 km grid cell (Chalhuanca: mean ± s.d.: 12.9 ± 11.5; Rio Apurimac: 12.5 ± 12.2; Wilcoxon test, *W* = 838, *p* = 0.6) or in the percentage of surveyed communities reporting animals bitten by bats (Chalhuanca: 71% (36/51 communities) and Rio Apurimac: 78% (92/118); electronic supplementary material, figure S4). The two valleys had equivalent prevalence of bat bites (*W* = 267, *p* = 0.8) and the presence/absence of bites (linear model with binomial error distribution controlling for the number of towns and the number of inspections per cell: valley effect, *t*-value_1,44_ = 0.74, *p* = 0.4). Together these data suggest widespread presence of bats in each valley with similar distributions of communities and survey effort.

### (c) Spatial forecasts of cross-species transmission to livestock

Our questionnaires confirmed the presence of vampire bats (as incidence of bat bites) ahead of advancing wavefronts, which will enable the spread of VBR into currently rabies-free parts of each valley (figure 5*a*). Moreover, only 25% (Chalhuanca) and 3% (Rio Apurimac) of respondents knew that bats could transmit rabies and fewer than half (47%: Chalhuanca and 40%: Rio Apurimac) knew the appropriate government agency for reporting rabies. Only 1 out of 90 farmers had vaccinated any animals against rabies.

Given the presence of vampire bats and the low preparedness for outbreaks ahead of wavefronts, we used our spatial models to forecast dates of rabies arrival to currently uninfected areas. The predictive power of our models was supported by our within-data validation. The true arrival of rabies fell within the predicted CIs in all outbreaks (electronic supplementary material, figure S3), with an average difference of 6.4 and 3.3 months between the predicted and actual arrival dates in Chalhuanca and Rio Apurimac, respectively (*n* = 5 and 7 hexagonal cells to predict, respectively). By forward projecting our full models, we predict VBR arrival to vampire bat populations in six currently uninfected districts by the end of 2015, 13 by 2016 and 22 by 2017 in the two valleys combined (figure 5*a*; electronic supplementary material, table S1).

## 4. Discussion

Using a high-resolution spatiotemporal dataset of rabies outbreaks, we illustrate the power of combining sentinel animal data from national surveillance systems with statistical models to predict the time and place of future cross-species transmissions of bat viruses. In Peru, we observe a recent doubling of VBR outbreaks in livestock, which is associated with spatial expansions of the virus into previously uninfected bat populations. In the region of the country where most outbreaks occur, the expansion forms multiple independent waves of infection that are travelling at consistent rates and trajectories towards communities where we document high contact rates between bats and unvaccinated livestock and poor knowledge of rabies. Widespread invasions into new areas and increases in the burden to human and animal health reveal VBR as an emerging rather than an enzootic zoonosis and mandate new strategies for rabies control in Latin America.

The consistent detection of VBR in one previously uninfected district per month over the last 12 years is an alarming signal of the growing human and animal health threat posed by VBR. The trigger(s) of these expansions are uncertain, but they are occurring throughout the country in transition zones between the Amazon and Andes and in valleys within the Andes (figure 3). One possibility is that growing livestock populations could have increased the size or connectivity of vampire bat populations, facilitating viral introduction [5]. However, livestock census data from 1994 and 2012 indicate no change in livestock densities in the newly infected districts (Peruvian National Livestock Census, CENAGRO). Another hypothesis is that rising temperatures due to climate change could expand bat populations and thus VBR to higher elevations [17]. Indeed, our GLMM shows that VBR is spreading on average to higher elevations. Importantly however, expansions occurred in areas well below the maximum elevation of VBR in other parts of the Peru (3600 mts), and the elevation of outbreaks did not increase in high elevation regions where VBR was enzootic throughout the time series (i.e. Apurimac and Ayacucho; figure 3). Thus, we argue that the emergence of VBR in these regions is caused not by climate-driven changes in the vampire bat distribution, but instead an epidemiological process of protracted viral invasion of a relatively new virus in vampire bat populations, a hypothesis supported by molecular clock estimates of the most recent common ancestor of vampire bat rabies [26]. In further support for the slow invasion hypothesis, the travelling waves in the two focal valleys of AAC were initiated only in the last few years despite molecular evidence that VBR was present in northwestern parts of the region since the early 1970s and the evidence from our questionnaire study that vampire bats are already present in rabies-free parts of each valley [14] (figure 5*a*).

The slow wavefront speeds that we observe in vampire bats relative to other rabies reservoirs (e.g. 30–100 km yr^−1^ in raccoons) and their low variability is more consistent with short distance dispersal of infected bats than invasion driven by rare long distance flights [27]. Indeed, although translocation experiments show that vampire bats are physiologically capable of long distance movements, home range sizes are typically <10 km^2^ [9,24,28]. One remaining question is why waves are travelling at different rates in different valleys. Different patterns of between-colony bat movements in each valley driven by underlying differences in the distribution of bat colonies could influence wave speeds. However, valleys had similar distributions of farms and intensities of bites (electronic supplementary material, figure S5), suggesting that long distance dispersal of VBR is unlikely to be a limiting factor in either valley. Anthropogenic disturbances, particularly the intensity of bat culls, could also conceivably influence viral spread. On the one hand, culls could reduce the probability of dispersal by an infected bat by reducing infection prevalence or competition-driven dispersal. On the other hand, dispersal by survivors of culls could facilitate viral invasion into uninfected areas. A similar example of disturbance-driven pathogen spread was observed in the UK, where disruption of badger territorial boundaries by culling facilitated the spatial spread of bovine tuberculosis [29]. Finally, differences in wavefront speeds could potentially arise if viral strains with different infection phenotypes circulate in each valley. However, previous work showed that only one geographically isolated viral lineage has circulated in the AAC since the early 1970s [14]. Therefore, we suspect that the waves described here have a common evolutionary origin, and recent evolution is unlikely to explain the observed variation in wavefront speeds.

In the absence of intervention, the arrival of VBR to currently uninfected areas will cause considerable livestock mortality, major economic losses and a new public health threat to farmers who handle infected bats or livestock. It is difficult to quantify the real impact of these viral invasions on agriculture, because under-reporting of VBR is thought to be significant but remains unquantified in most areas of Latin America, precluding estimation of the true burden of the disease on livestock [30]. In the two AAC valleys alone, we forecast that over 11 600 small-scale farms, containing at least 339 000 livestock (93 715 cows, 215 707 sheep, 15 690 goats and 13 774 pigs) will newly be at risk of rabies in the next 3 years. Vaccination of these animals would cost at least US$373 000 per year (US$1.1 per vaccine); a significant financial burden to small-scale farmers who rely on livestock sales to pay for house maintenance and childhood education and for local governments that have not previously been affected by rabies. The high cost of vaccines, together with low local knowledge of rabies indicated by our questionnaires, presents a major challenge to overcome with epizootic invasion impending.

To aid planning of interventions, we provide a forecast of rabies arrival dates to presently uninfected areas. Like all predictive models, our projection uncertainty increases further into the future; thus, it will be useful to update models as additional data become available. Nevertheless, the consistency of wavefront speeds, even after considering uncertainty in origin locations, and the ability of our model to successfully predict known dates of rabies arrival provide confidence in our forecasts. Although our phenomenological models of rabies spread account for habitat heterogeneity using a least-cost distance algorithm, other methods exist for estimating wavefront speed. Statistical methods such as trend surface analysis are used over larger spatial and time scales than our data, where outbreaks are distributed along relatively short valleys. Spatially explicit mechanistic models have also been widely used to model rabies spread in carnivores in heterogeneous landscapes (e.g. [27,31]). Unfortunately, the dispersal distances of infected bats and the sizes of colonies are unknown, so parameterization of such a model for VBR would not be straightforward. Moreover, without additional data, such models would not be expected to alter our mean predictions. We therefore argue our approach is ideally suited to inform rapid responses in a public health and veterinary emergency.

Travelling waves have been identified in several other wildlife zoonoses (e.g. Ebola virus [23], rabies in raccoons [32] and plague in rodents [33]). The waves of VBR that we identify are important, because they are occurring in real time and are predictable in speed and trajectory, creating a powerful opportunity to direct the distribution of vaccines and educational campaigns to areas where infection is imminent (electronic supplementary material, table S1). Our results further open new possibilities for the control of a bat-borne zoonosis in its reservoir. While interventions such as culling have historically focused on enzootic areas, our findings enable targeting of healthy bat populations before viral invasion. Such a strategy was attempted in Argentina in the 1970s, but even with a dramatic 95% reduction of vampire bat populations using cyanide poisoning, the epizootic advanced [34]. This failure could reflect an absence of natural geographical barriers allowing the virus to circumnavigate the intervention zone or the challenges of using population reduction to control a pathogen with frequency-dependent transmission [9,10,35]. An appealing alternative would be to increase the proportion of immunized bats while maintaining the age structure and relatively sedentary dispersal behaviour of vampire bats. A transmissible gel-based topical rabies vaccine was effective in captive vampire bats but has never been released in wild populations [36]. Vaccination ahead of the epizootic front could be particularly effective when paired with natural geographical barriers such as the high Andean peaks that occur in the AAC.

This study demonstrates the power of animal health surveillance systems to generate high-resolution insights into the spatiotemporal dynamics of zoonotic viruses that would probably be impossible to detect relying on studies within a wildlife reservoir alone. The travelling waves that we detected in Peru directly inform management of viral spillover from bats by providing recommendations for where and when livestock should be vaccinated and educational campaigns should be carried out, while creating a unique opportunity to trail experimental interventions in bat populations to block spatially replicated advancing epizootics.

## Supplementary Material

electronic supplementary material

## Data accessibility

Data used in this study were provided by the Servicio Nacional de Sanidad Agraria (SENASA) of Peru, publicly available online at http://www.senasa.gob.pe/senasa/reportes-epidemiologicos-semanales. Bat bite data may be requested from the Apurimac Regional Project for Rabies Control.
