# Supplementary material for: Spatial expansions and travelling waves of rabies in vampire bats
Source: Proc Biol Sci. 2016 Jun 15;283(1832):20160328. doi: 10.1098/rspb.2016.0328 (PMC4920313; doi:10.1098/rspb.2016.0328)
Supplement: electronic supplementary material [file rspb20160328supp1.pdf]

Electronic Supplementary Materials

Spatial Expansions and Travelling Waves of Rabies in Vampire Bats

Julio A. Benavides, William Valderrama and Daniel G. Streicker

1- Datasets

a- Outbreak reports from SENASA

Since we focused on the distribution of outbreaks rather than the total number of cases, we analyzed only the first positive report associated with each outbreak or the first negative report (if all suspected cases from that farm were rabies negative) when multiple animals were reported sick or dead. Information for each outbreak included species infected, date of disease onset, the district where the outbreak occurred (Peru is divided into regions, provinces, districts and towns), and GPS data when available from the farm or community where the outbreak occurred (N = 914 outbreaks). We excluded data from two regions on the coast of Peru (Lima and Ancash) that reported small numbers of unconfirmed outbreaks and 23 outbreaks from the region of Puno, where dog rabies causes most livestock infections [1].

b- Questionnaires on bites ahead of travelling waves

Using a standardized questionnaire, we asked farmers whether their animals had been bitten by vampire bats in the last year; whether they knew that vampire bats can transmit rabies; whether they knew that SENASA was the relevant authority for reporting rabies; and if they vaccinated their animals against rabies. Questionnaires were performed in Quechua or Spanish by a local assistant. Procedures were approved by the Ethics Committee of the College of Veterinary Science, University of Glasgow. Written and signed informed consent approval was obtained from each farmer who participated in the study.

2- Least cost distance model

Least-cost distances were calculated using the *gdistance* package of R, setting resistance values to 1 and 10000 below and above 3,600m, respectively. The *transition* function was used to create a least-cost distance across a conductance map, with 8 possible path directions. Distances were corrected to account for geographical projection using the *geoCorrection* function. The

costDistance function was used to calculate the distance from the origin of each wave to the center of each hexbin.

3- Sensitivity analysis

a- The influence of the index origin on wavefront speeds

To assess the sensitivity of wavefront speeds and R^2 to the inferred location of index outbreaks, we performed a bootstrap analysis using the total number of outbreaks *N* [2]. This analysis consisted in four consecutive steps: (1) randomly sampling *N* outbreaks, allowing the same outbreak to be sampled multiple times (*i.e.* bootstrapping with replacement) using the *sample* function of R; (2) randomly sampling 5000 different plausible origins for each wave; (3) for each of those 5000 origins, calculating the slope and R^2 of the regression between the least-cost distance to the origin and the time of disease arrival; (4) selecting the slope of the regression with the highest R^2 for each origin. This procedure was repeated 200 times for each valley, and the range of both the speed of the wave (1/slope of the regression) and its associated R^2 were estimated. The distributions of the speed of the wave and R^2 obtained are shown in figure S1.

b- The influence of the elevation threshold on viral spread

This analysis was the same as the one described above, except for step (1). This step was replaced by randomly selecting an elevation threshold from a uniform distribution ranging from 3500 to 4500 meters using the *runif* function in R. We focus our sensitivity analysis on elevations above 3600 meters because clear evidence of bat presence and viral circulation below this limit which implies the absence of any impassable threshold (figure 3, figure 5). In both valleys, the estimated speed of the wave and R^2 declined slightly with the elevation threshold (figure S2). However, all estimated values remained within the 95% CI of the estimation using 3600 meters, the assumed value in our main analysis. Thus, the estimation of the wave speed was largely insensitive to the elevation threshold. The negative correlation between wavefront speeds and R^2 and the elevation threshold is explained by longer least-cost distances when using a smaller elevation threshold because there are more elevation obstacles in the landscape.

4- Cross-validation analysis

We validated the predictions of outbreak arrival dates to a particular location ahead of the wave using the following procedure. First, we estimated the most likely origin of the wave by

maximising the R^2 of the linear regression using the first 75% of outbreak locations. We then predicted the month of arrival for the remaining outbreak locations in the wave front (5 and 7 hexagonal cells to predict in Chalhuanca and Rio Apurimac, respectively) using the *predict* function in R. Finally, we calculated the averaged difference between the predicted and arrival date. All true arrival dates from the test dataset occurred within the 95% CI of the predicted arrival dates (figure S1). Similar results were obtained when using half of the data to predict the other half (50% training, 50% test set); all arrival dates of the test set occurred within the 95% CI of the predicted arrival dates.

References

1. Condori-Condori R.E., Streicker D.G., Cabezas-Sanchez C., Velasco-Villa A. 2013 Enzootic and epizootic rabies associated with vampire bats, Peru. *Emerg Infect Dis* **19**(9), 1463.
2. Mooney C.Z., Duval R.D., Duval R. 1993 *Bootstrapping: A nonparametric approach to statistical inference*, Sage.

Supplementary Figures and Tables

Figure S1. Sensitivity of wavefront speeds to the inferred wave origins. The distribution of the estimated wave speeds (left) and their associated R^2 (right) from the bootstrap procedure are shown for Chalhuanca (top plots) and Rio Apurimac (bottom plots).

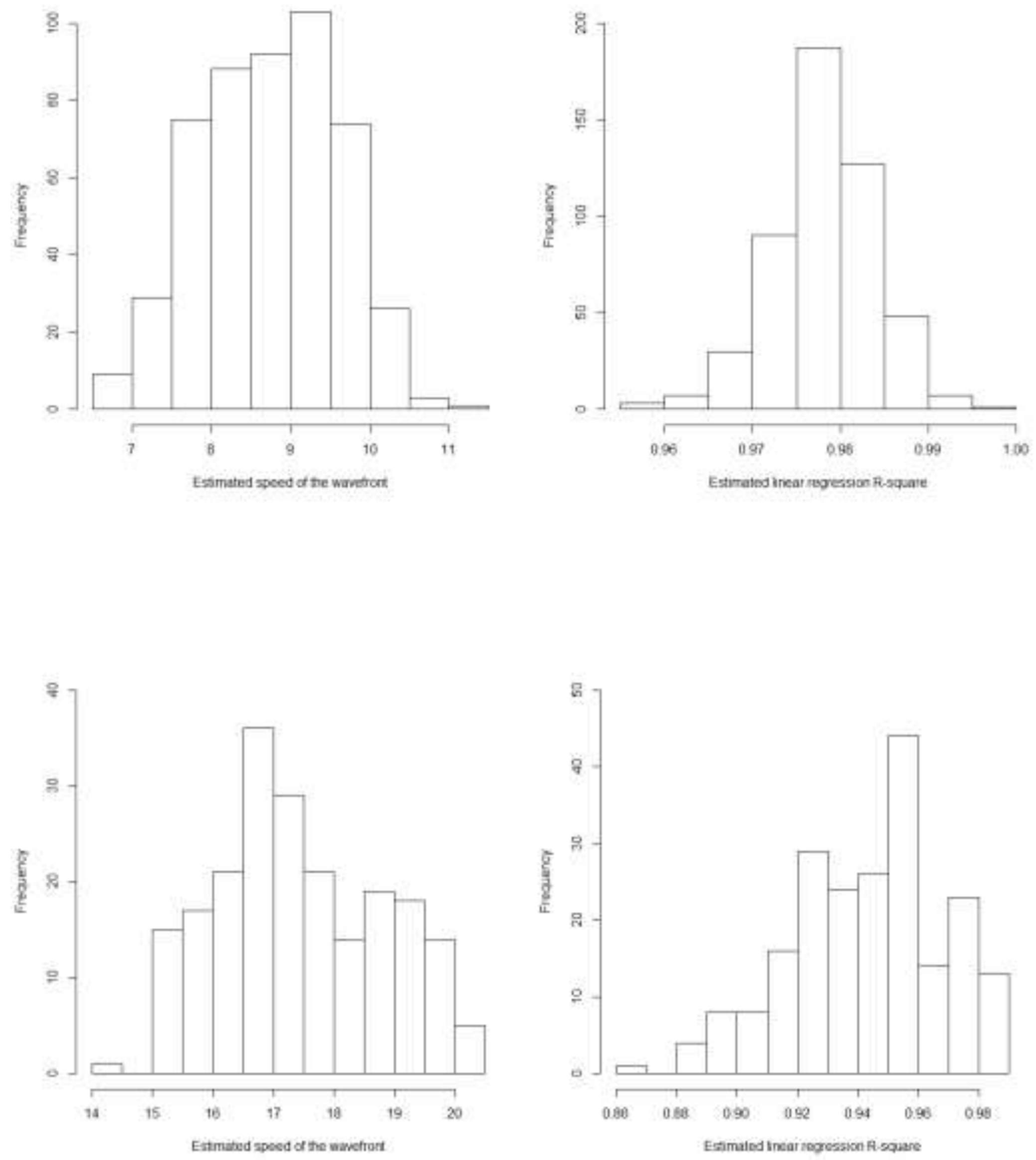

Figure S2. Results of sensitivity analysis of wavefront speeds to the elevation threshold. The relationship between randomly chosen elevation thresholds and their estimated wave speed (left) and their associated R^2 (right) are shown for Chalhuanca (top plots) and Rio Apurimac (bottom plots).

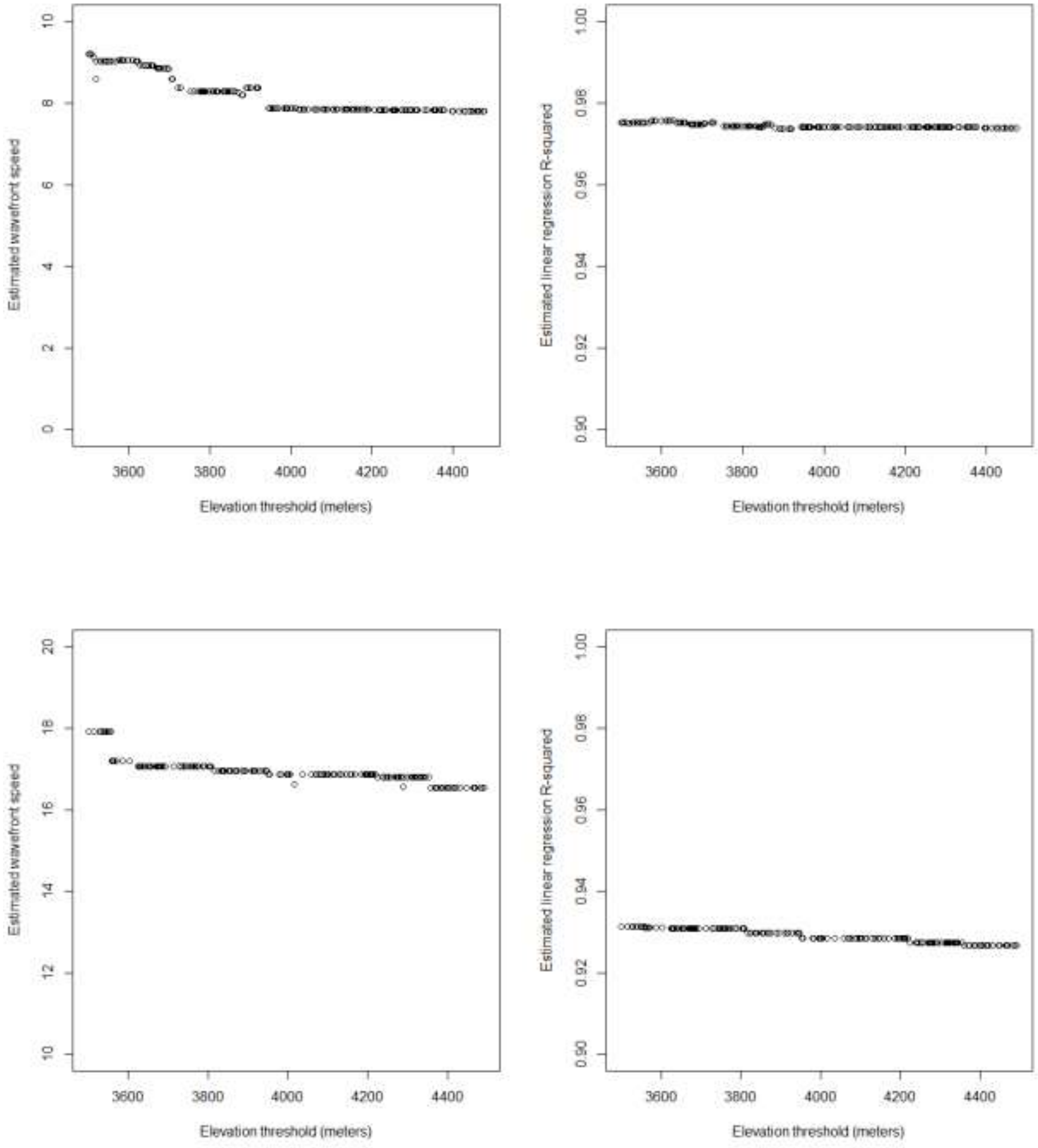

Figure S3. Cross-validation of predictions on the arrival of vampire bat rabies to new regions. Vertical lines show the 95% CI of predicted outbreak locations of the test data set (in black points) in Chalhuanca (left) and Rio Apurimac (right). Open points are data used in the training model.

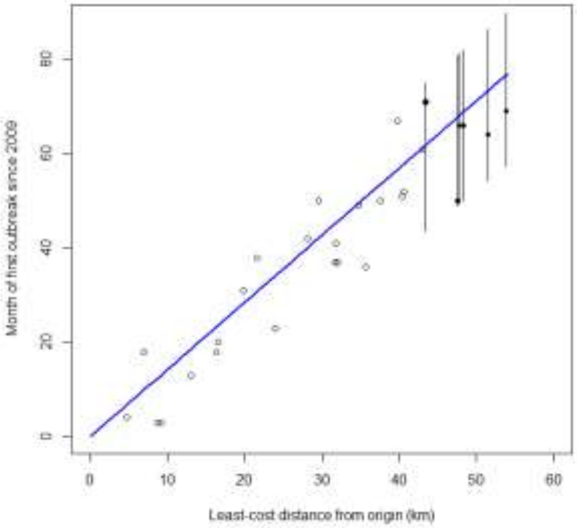

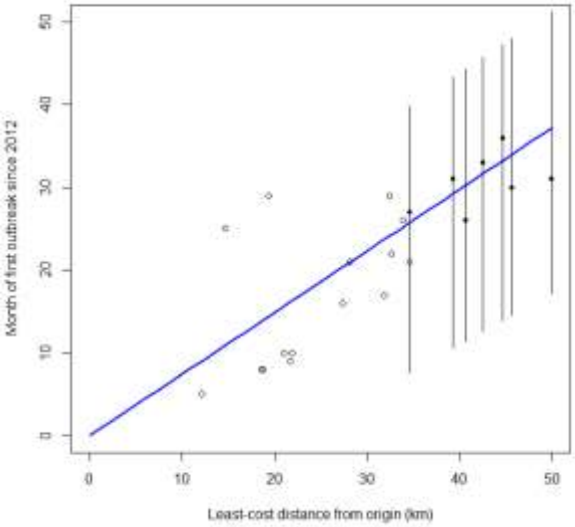

Figure S4. Spatial distribution of farms reporting animals with bites in valleys where rabies is spreading as a wave. (a) Grey dots correspond to the center of 5km radius hexagonal cells, with the size of the dot proportional to the number of towns in that cell (range: 1-60). Blue (Chalhuanca valley) and red (Rio Apurimac valley) dots show cells in which animals were being bitten by vampire bats. Yellow dots show cells were none of the inspected animals were bitten. Only towns within the wave spread limits as of 2014 are shown. (b) Distributions of the number of towns per cell, and the proportion of farms with bitten animals in both valleys (inset). Vertical lines show the averaged value of each distribution. Blue and red colors show Chalhuanca and Rio Apurimac valley's distribution respectively.

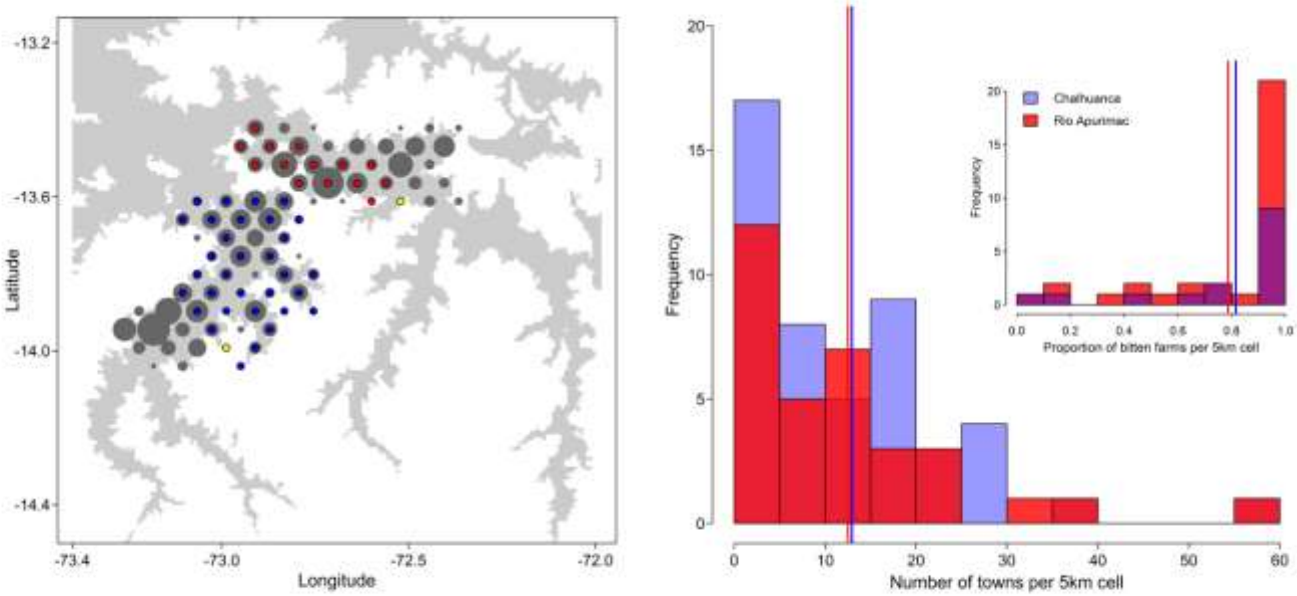

Figure S5. Reporting patterns of other notifiable livestock diseases and rabies. (a) Number of outbreaks for the 11 most common notifiable diseases of livestock in Peru. (b) Number of outbreaks (or governmental districts reporting, inset) of each year for the 56 diseases affecting livestock (excluding rabies) reported to the national surveillance system of SENASA. Lines show the predicted values using the loess function in R.

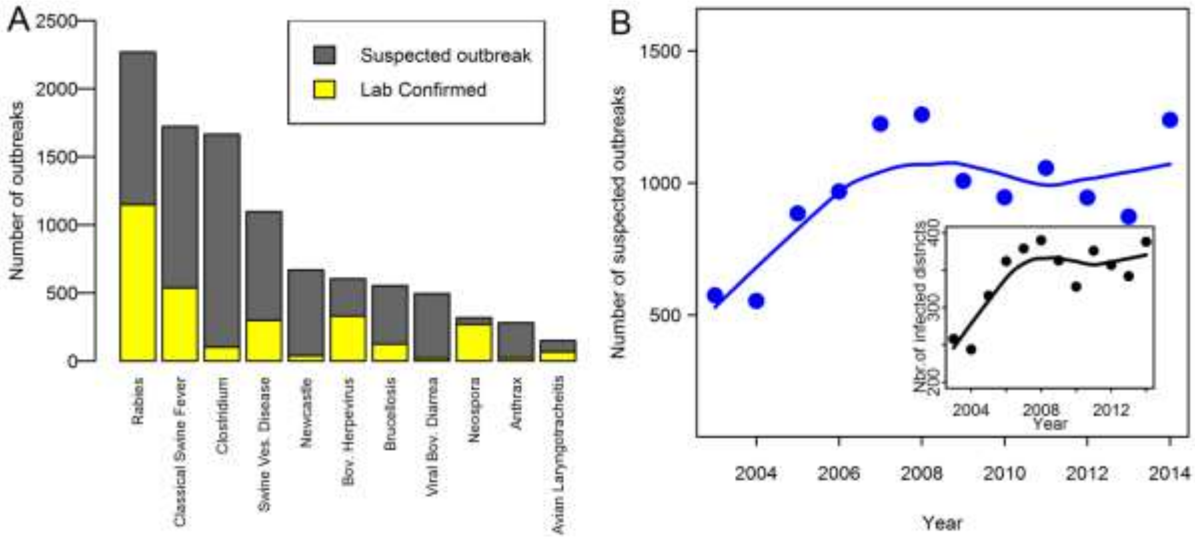

Figure S6. Number of outbreaks in each region by month since 2003. Andean and Amazonian regions are colored in blue and red, respectively.

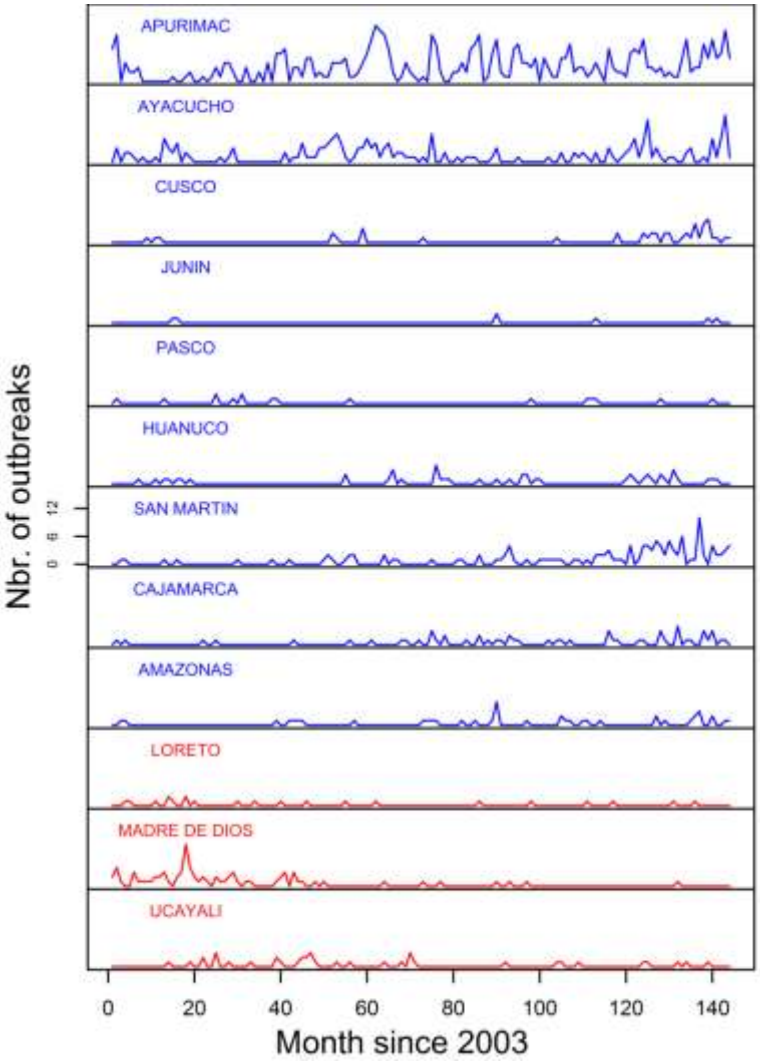

Table S1. Month of predicted VBR arrival to towns ahead of each wave. The date of VBR arrival between 2015 and 2017 was predicted for each town under 3600 meters recorded in the national census. Low and high months of predicted arrival are 95% prediction intervals, estimated from the regression models from Figure 4 using the *predict.lm* function of R. Predicted arrivals before 2014 in the low confidence interval were assigned to 01/2015. We note that dates of predicted arrival are based on a simple landscape heterogeneity model of least cost paths from the predicted origin below 3600 meters, which could be overestimates if unidentified landscape barriers exist. Our predictions will lose accuracy if the speed of the epizootic changes and later dates should be re-estimated as new data become available.

Town	District	Province	Department	Longitude	Latitude	Low Prediction Date	Average Prediction Date	High Prediction Date
ATIZO	EL ORO	ANTABAMBA	APURIMAC	-73.072	-14.2047	8/2016	12/2017	4/2019
MISCAPATA	EL ORO	ANTABAMBA	APURIMAC	-73.0713	-14.204	8/2016	12/2017	4/2019
ACCOLLANCCA	HUAYLLO	AYMARAES	APURIMAC	-73.2349	-14.0877	01/2015	2/2016	6/2017
ANTAPAMPA	HUAYLLO	AYMARAES	APURIMAC	-73.2731	-14.133	6/2015	10/2016	2/2018
APACA	HUAYLLO	AYMARAES	APURIMAC	-73.2157	-14.0462	01/2015	7/2015	10/2016
CAINIPAMPA	HUAYLLO	AYMARAES	APURIMAC	-73.2734	-14.1242	5/2015	9/2016	1/2018
CANCAUPATA	HUAYLLO	AYMARAES	APURIMAC	-73.2718	-14.1264	6/2015	10/2016	2/2018
CCAPEC PAMPA	HUAYLLO	AYMARAES	APURIMAC	-73.2289	-14.0584	01/2015	10/2015	2/2017
CHAUCHANI	HUAYLLO	AYMARAES	APURIMAC	-73.2742	-14.1287	6/2015	10/2016	2/2018
CHILLCAPATA	HUAYLLO	AYMARAES	APURIMAC	-73.2273	-14.0869	01/2015	2/2016	5/2017
CHISMAPAMPA	HUAYLLO	AYMARAES	APURIMAC	-73.2914	-14.1318	7/2015	11/2016	3/2018
CHULLU PAMPA / CHALLHUAPAMPA	HUAYLLO	AYMARAES	APURIMAC	-73.2034	-14.034	01/2015	5/2015	8/2016
CONCHA PALLANA	HUAYLLO	AYMARAES	APURIMAC	-73.2178	-14.066	01/2015	10/2015	1/2017
CRUZ PATA	HUAYLLO	AYMARAES	APURIMAC	-73.2739	-14.1346	8/2015	11/2016	3/2018
HUAYQUIPA	HUAYLLO	AYMARAES	APURIMAC	-73.2365	-14.081	01/2015	1/2016	5/2017
IHUAYLLO	HUAYLLO	AYMARAES	APURIMAC	-73.2679	-14.1334	8/2015	11/2016	3/2018
MOLINO PAMPA	HUAYLLO	AYMARAES	APURIMAC	-73.2851	-14.1271	7/2015	11/2016	3/2018
MOTCANI	HUAYLLO	AYMARAES	APURIMAC	-73.2732	-14.127	6/2015	10/2016	2/2018
OCCO	HUAYLLO	AYMARAES	APURIMAC	-73.2115	-14.0378	01/2015	5/2015	9/2016
OLLUCOPATA	HUAYLLO	AYMARAES	APURIMAC	-73.2319	-14.0885	01/2015	2/2016	5/2017
PATIYO PAMPA	HUAYLLO	AYMARAES	APURIMAC	-73.2594	-14.1059	2/2015	6/2016	10/2017
PICHUS	HUAYLLO	AYMARAES	APURIMAC	-73.1918	-14.0166	01/2015	1/2015	4/2016
PUCAJASA	HUAYLLO	AYMARAES	APURIMAC	-73.2622	-14.0939	1/2015	5/2016	9/2017
PUCARA	HUAYLLO	AYMARAES	APURIMAC	-73.2846	-14.131	7/2015	11/2016	3/2018
PUCRO	HUAYLLO	AYMARAES	APURIMAC	-73.2818	-14.1296	7/2015	11/2016	3/2018

PUENTE HUAYQUIPA	HUAYLLO	AYMARAES	APURIMAC	-73.2554	-14.0748	01/2015	1/2016	4/2017
RETAMANI	HUAYLLO	AYMARAES	APURIMAC	-73.2797	-14.1156	4/2015	8/2016	12/2017
SALLALLE	HUAYLLO	AYMARAES	APURIMAC	-73.2285	-14.0784	01/2015	12/2015	4/2017
SAN ANTONIO	HUAYLLO	AYMARAES	APURIMAC	-73.2733	-14.1246	5/2015	9/2016	1/2018
SOCCOS	HUAYLLO	AYMARAES	APURIMAC	-73.2822	-14.1253	7/2015	11/2016	3/2018
TAMICA	HUAYLLO	AYMARAES	APURIMAC	-73.2816	-14.1295	7/2015	11/2016	3/2018
AMOCA	JUSTO APU SAHUARAURA	AYMARAES	APURIMAC	-73.1714	-14.1122	01/2015	2/2016	6/2017
CCELLAICHUMPE / CCELLAYCHUMPE	JUSTO APU SAHUARAURA	AYMARAES	APURIMAC	-73.1827	-14.15	5/2015	9/2016	12/2017
CCESHUA	JUSTO APU SAHUARAURA	AYMARAES	APURIMAC	-73.163	-14.1514	5/2015	9/2016	12/2017
CCOLLCCAPATA	JUSTO APU SAHUARAURA	AYMARAES	APURIMAC	-73.1777	-14.1683	7/2015	11/2016	3/2018
CHECCASA	JUSTO APU SAHUARAURA	AYMARAES	APURIMAC	-73.1966	-14.1277	2/2015	6/2016	10/2017
CHINCHINÍA	JUSTO APU SAHUARAURA	AYMARAES	APURIMAC	-73.1883	-14.1664	6/2015	10/2016	2/2018
HACIENDA CHALLHUAPAMPA	JUSTO APU SAHUARAURA	AYMARAES	APURIMAC	-73.1869	-14.0754	01/2015	10/2015	2/2017
HACIENDA PICHUS	JUSTO APU SAHUARAURA	AYMARAES	APURIMAC	-73.191	-14.0501	01/2015	6/2015	10/2016
HUACCASA	JUSTO APU SAHUARAURA	AYMARAES	APURIMAC	-73.1831	-14.1601	6/2015	10/2016	2/2018
MITAMA	JUSTO APU SAHUARAURA	AYMARAES	APURIMAC	-73.1906	-14.1684	8/2015	11/2016	3/2018
PACSICA	JUSTO APU SAHUARAURA	AYMARAES	APURIMAC	-73.1604	-14.1447	3/2015	7/2016	11/2017
PALLJA	JUSTO APU SAHUARAURA	AYMARAES	APURIMAC	-73.2006	-14.1286	3/2015	6/2016	10/2017
PICHIHUA	JUSTO APU SAHUARAURA	AYMARAES	APURIMAC	-73.1739	-14.1482	3/2015	7/2016	11/2017
PISCOBAMBA	JUSTO APU SAHUARAURA	AYMARAES	APURIMAC	-73.2024	-14.1222	1/2015	5/2016	9/2017

PUMAHUACHO	JUSTO APU SAHUARAURA	AYMARAES	APURIMAC	-73.1772	-14.1163	01/2015	2/2016	6/2017
PUQUIO	JUSTO APU SAHUARAURA	AYMARAES	APURIMAC	-73.1658	-14.1599	6/2015	10/2016	2/2018
SURUCRO	JUSTO APU SAHUARAURA	AYMARAES	APURIMAC	-73.1884	-14.1493	4/2015	8/2016	12/2017
UMACCATA	JUSTO APU SAHUARAURA	AYMARAES	APURIMAC	-73.1808	-14.1524	5/2015	9/2016	12/2017
VISCACHAYOC	JUSTO APU SAHUARAURA	AYMARAES	APURIMAC	-73.169	-14.1582	4/2015	8/2016	12/2017
CNANIO	POCOHUANCA	AYMARAES	APURIMAC	-73.1192	-14.2221	5/2016	9/2017	1/2019
ALLPAMACHAY	POCOHUANCA	AYMARAES	APURIMAC	-73.0935	-14.2104	5/2016	9/2017	1/2019
CASCABAMBA	POCOHUANCA	AYMARAES	APURIMAC	-73.0785	-14.2198	8/2016	12/2017	4/2019
CCACHORAY	POCOHUANCA	AYMARAES	APURIMAC	-73.0799	-14.2065	7/2016	11/2017	3/2019
CHANTA	POCOHUANCA	AYMARAES	APURIMAC	-73.1174	-14.2254	6/2016	10/2017	2/2019
CHILLICO	POCOHUANCA	AYMARAES	APURIMAC	-73.0912	-14.1998	5/2016	9/2017	1/2019
HORNOBAMBA	POCOHUANCA	AYMARAES	APURIMAC	-73.1151	-14.2083	3/2016	7/2017	11/2018
HUANCAPAMPA	POCOHUANCA	AYMARAES	APURIMAC	-73.0766	-14.2108	7/2016	11/2017	4/2019
HUAYCALO	POCOHUANCA	AYMARAES	APURIMAC	-73.0825	-14.213	7/2016	11/2017	4/2019
POCOHUANCA	POCOHUANCA	AYMARAES	APURIMAC	-73.087	-14.2185	6/2016	11/2017	3/2019
PUENTE	POCOHUANCA	AYMARAES	APURIMAC	-73.125	-14.2034	2/2016	6/2017	10/2018
SALLIPUCRO	POCOHUANCA	AYMARAES	APURIMAC	-73.0814	-14.2033	7/2016	11/2017	3/2019
TASCANCCOCHA	POCOHUANCA	AYMARAES	APURIMAC	-73.0895	-14.2154	6/2016	10/2017	2/2019
TIAPARO	POCOHUANCA	AYMARAES	APURIMAC	-73.0964	-14.1764	4/2016	8/2017	12/2018
CALAMASMA	SANAYCA	AYMARAES	APURIMAC	-73.3205	-14.1931	7/2016	11/2017	3/2019
CHACAPUENTE	SANAYCA	AYMARAES	APURIMAC	-73.3031	-14.142	11/2015	3/2017	7/2018
CHIRIYACU	SANAYCA	AYMARAES	APURIMAC	-73.3376	-14.1527	2/2016	6/2017	10/2018
CHIUJA	SANAYCA	AYMARAES	APURIMAC	-73.3222	-14.1853	6/2016	10/2017	2/2019
CON ÍECYACU	SANAYCA	AYMARAES	APURIMAC	-73.3071	-14.1462	11/2015	3/2017	7/2018
HACIENDA HUAYARA	SANAYCA	AYMARAES	APURIMAC	-73.3481	-14.1633	3/2016	7/2017	12/2018

NINAYBAMBA	SANAYCA	AYMARAES	APURIMAC	-73.3166	-14.1531	12/2015	4/2017	8/2018
ACANI	SORAYA	AYMARAES	APURIMAC	-73.2933	-14.1741	2/2016	6/2017	10/2018
AHUA	SORAYA	AYMARAES	APURIMAC	-73.3227	-14.1706	3/2016	7/2017	11/2018
CANTO	SORAYA	AYMARAES	APURIMAC	-73.2959	-14.146	10/2015	2/2017	6/2018
CARAPAMPA	SORAYA	AYMARAES	APURIMAC	-73.2977	-14.1723	2/2016	6/2017	10/2018
CCARAHUATANI	SORAYA	AYMARAES	APURIMAC	-73.2891	-14.1705	1/2016	5/2017	9/2018
CCARCCANTA	SORAYA	AYMARAES	APURIMAC	-73.2883	-14.1459	10/2015	2/2017	6/2018
CHAYA	SORAYA	AYMARAES	APURIMAC	-73.3186	-14.1801	4/2016	8/2017	1/2019
CHILLICO	SORAYA	AYMARAES	APURIMAC	-73.2977	-14.1509	11/2015	3/2017	7/2018
CHIMPACCANTO	SORAYA	AYMARAES	APURIMAC	-73.2898	-14.139	8/2015	12/2016	4/2018
CHUSPIPATA	SORAYA	AYMARAES	APURIMAC	-73.2993	-14.1442	10/2015	2/2017	6/2018
CHUYCHO	SORAYA	AYMARAES	APURIMAC	-73.2952	-14.1663	1/2016	5/2017	9/2018
COCHAPAMPA	SORAYA	AYMARAES	APURIMAC	-73.2871	-14.168	1/2016	5/2017	9/2018
COMUNI	SORAYA	AYMARAES	APURIMAC	-73.3024	-14.1479	11/2015	3/2017	7/2018
HUMAPAMPA	SORAYA	AYMARAES	APURIMAC	-73.3169	-14.1625	2/2016	6/2017	10/2018
IHUACAY	SORAYA	AYMARAES	APURIMAC	-73.2843	-14.17	1/2016	5/2017	9/2018
ISLA PAMPA	SORAYA	AYMARAES	APURIMAC	-73.2792	-14.1721	2/2016	6/2017	10/2018
JACHURA	SORAYA	AYMARAES	APURIMAC	-73.2967	-14.1754	3/2016	7/2017	11/2018
LLAUCAHUACHO	SORAYA	AYMARAES	APURIMAC	-73.2965	-14.1541	11/2015	3/2017	7/2018
MISQUILLACO / MISQUIYACU	SORAYA	AYMARAES	APURIMAC	-73.3001	-14.1642	1/2016	5/2017	9/2018
PACAYCA	SORAYA	AYMARAES	APURIMAC	-73.306	-14.1493	11/2015	3/2017	7/2018
PULLPUNI	SORAYA	AYMARAES	APURIMAC	-73.2949	-14.1817	3/2016	7/2017	11/2018
SORAYA	SORAYA	AYMARAES	APURIMAC	-73.3149	-14.1649	1/2016	6/2017	10/2018
ACIRUMA	TAPAIRIHUA	AYMARAES	APURIMAC	-73.1393	-14.0963	01/2015	1/2016	5/2017
ACOBAMBA	TAPAIRIHUA	AYMARAES	APURIMAC	-73.1321	-14.1287	3/2015	7/2016	11/2017
ANHUACCHAY / ANCCACCHUAY	TAPAIRIHUA	AYMARAES	APURIMAC	-73.1256	-14.1867	11/2015	3/2017	7/2018

ANOCCARA / ACOCCARA	TAPAIRIHUA	AYMARAES	APURIMAC	-73.1413	-14.1444	5/2015	9/2016	1/2018
AYLLASANA	TAPAIRIHUA	AYMARAES	APURIMAC	-73.1466	-14.111	01/2015	3/2016	7/2017
CCOLLPA	TAPAIRIHUA	AYMARAES	APURIMAC	-73.1555	-14.1395	3/2015	7/2016	10/2017
CCORIRAY	TAPAIRIHUA	AYMARAES	APURIMAC	-73.1684	-14.0922	01/2015	11/2015	3/2017
CHIHUA	TAPAIRIHUA	AYMARAES	APURIMAC	-73.1842	-14.0415	01/2015	4/2015	7/2016
CHILCAPATA	TAPAIRIHUA	AYMARAES	APURIMAC	-73.1352	-14.1565	6/2015	10/2016	2/2018
CHIUCHIN / CHUCHIN	TAPAIRIHUA	AYMARAES	APURIMAC	-73.1288	-14.142	5/2015	9/2016	1/2018
CHOQUEMARCA	TAPAIRIHUA	AYMARAES	APURIMAC	-73.135	-14.1523	6/2015	10/2016	2/2018
CHUCHIMPATA	TAPAIRIHUA	AYMARAES	APURIMAC	-73.1346	-14.0772	01/2015	12/2015	3/2017
COTABAMBA	TAPAIRIHUA	AYMARAES	APURIMAC	-73.1267	-14.1256	3/2015	7/2016	11/2017
HUACCAMOLLE	TAPAIRIHUA	AYMARAES	APURIMAC	-73.156	-14.1375	3/2015	7/2016	10/2017
HUACLLAPATA	TAPAIRIHUA	AYMARAES	APURIMAC	-73.118	-14.1395	5/2015	9/2016	12/2017
HUANQUIRNA / HUANQUIRINA	TAPAIRIHUA	AYMARAES	APURIMAC	-73.1458	-14.141	3/2015	7/2016	11/2017
HUAYAO	TAPAIRIHUA	AYMARAES	APURIMAC	-73.1332	-14.1431	5/2015	9/2016	1/2018
LAIME	TAPAIRIHUA	AYMARAES	APURIMAC	-73.1251	-14.1123	3/2015	7/2016	11/2017
LAYME	TAPAIRIHUA	AYMARAES	APURIMAC	-73.126	-14.1123	3/2015	7/2016	11/2017
LLIPCCA	TAPAIRIHUA	AYMARAES	APURIMAC	-73.1397	-14.0963	01/2015	1/2016	5/2017
LUYCHUPATA	TAPAIRIHUA	AYMARAES	APURIMAC	-73.1554	-14.14	3/2015	7/2016	10/2017
MAZUPAMPA / MAZOPAMPA	TAPAIRIHUA	AYMARAES	APURIMAC	-73.1308	-14.1927	1/2016	5/2017	9/2018
MOCHOCCO	TAPAIRIHUA	AYMARAES	APURIMAC	-73.1794	-14.0896	01/2015	11/2015	2/2017
MONTE	TAPAIRIHUA	AYMARAES	APURIMAC	-73.1689	-14.1036	01/2015	1/2016	4/2017
PACCHANTAY	TAPAIRIHUA	AYMARAES	APURIMAC	-73.133	-14.1704	9/2015	1/2017	5/2018
PALLCCORA	TAPAIRIHUA	AYMARAES	APURIMAC	-73.1481	-14.0944	01/2015	1/2016	5/2017
PATAPATA	TAPAIRIHUA	AYMARAES	APURIMAC	-73.1274	-14.1603	8/2015	12/2016	4/2018
PICHIRIJAY	TAPAIRIHUA	AYMARAES	APURIMAC	-73.1423	-14.0845	01/2015	12/2015	3/2017
POCCOCHIHUA	TAPAIRIHUA	AYMARAES	APURIMAC	-73.1432	-14.1405	3/2015	7/2016	11/2017

POTOCINO	TAPAIRIHUA	AYMARAES	APURIMAC	-73.1306	-14.1272	3/2015	7/2016	11/2017
QUISCHQUE	TAPAIRIHUA	AYMARAES	APURIMAC	-73.1345	-14.1608	7/2015	11/2016	3/2018
SALLALLUME	TAPAIRIHUA	AYMARAES	APURIMAC	-73.1412	-14.137	4/2015	8/2016	11/2017
SAN LUIS	TAPAIRIHUA	AYMARAES	APURIMAC	-73.1276	-14.1878	11/2015	3/2017	7/2018
SOCCO	TAPAIRIHUA	AYMARAES	APURIMAC	-73.1682	-14.1106	01/2015	2/2016	6/2017
TACAHUARA	TAPAIRIHUA	AYMARAES	APURIMAC	-73.1613	-14.1026	01/2015	1/2016	5/2017
TAMBO	TAPAIRIHUA	AYMARAES	APURIMAC	-73.1566	-14.0878	01/2015	11/2015	3/2017
TAPAIRIHUA	TAPAIRIHUA	AYMARAES	APURIMAC	-73.1405	-14.1413	4/2015	8/2016	11/2017
VIMPILLAY	TAPAIRIHUA	AYMARAES	APURIMAC	-73.1352	-14.1319	3/2015	6/2016	10/2017
APARAY	TORAYA	AYMARAES	APURIMAC	-73.2738	-14.0526	01/2015	12/2015	4/2017
ATUMPAMPA	TORAYA	AYMARAES	APURIMAC	-73.2931	-14.0699	1/2015	5/2016	8/2017
BUENAVISTA	TORAYA	AYMARAES	APURIMAC	-73.2975	-14.0621	01/2015	4/2016	8/2017
CANUA	TORAYA	AYMARAES	APURIMAC	-73.2994	-14.0391	01/2015	2/2016	6/2017
CCASAYA	TORAYA	AYMARAES	APURIMAC	-73.2874	-14.0304	01/2015	1/2016	4/2017
CHALLHUINI	TORAYA	AYMARAES	APURIMAC	-73.3057	-14.0612	1/2015	5/2016	9/2017
CHUCHIN	TORAYA	AYMARAES	APURIMAC	-73.2992	-14.0316	01/2015	2/2016	6/2017
CONDEBAMBA	TORAYA	AYMARAES	APURIMAC	-73.2833	-14.0459	01/2015	1/2016	4/2017
HACIENDA SAN FRANCISCO	TORAYA	AYMARAES	APURIMAC	-73.2505	-14.0605	01/2015	11/2015	3/2017
HUANCARIRCA	TORAYA	AYMARAES	APURIMAC	-73.3115	-14.0683	3/2015	7/2016	11/2017
HUANCAROPATA	TORAYA	AYMARAES	APURIMAC	-73.2659	-14.0385	01/2015	10/2015	1/2017
JASAYA	TORAYA	AYMARAES	APURIMAC	-73.2941	-14.0346	01/2015	2/2016	6/2017
LLAN ÍUPAMPA	TORAYA	AYMARAES	APURIMAC	-73.2761	-14.0473	9/2014	1/2016	4/2017
LLINQUE	TORAYA	AYMARAES	APURIMAC	-73.2987	-14.0059	11/2014	2/2016	6/2017
LUYCHOPUQUIO	TORAYA	AYMARAES	APURIMAC	-73.2994	-14.0916	2/2015	6/2016	9/2017
MAJAPATA	TORAYA	AYMARAES	APURIMAC	-73.333	-14.0317	3/2015	7/2016	11/2017
MAYOPAMPA	TORAYA	AYMARAES	APURIMAC	-73.2879	-14.0983	2/2015	6/2016	10/2017
MOLINO	TORAYA	AYMARAES	APURIMAC	-73.2937	-14.0366	11/2014	2/2016	6/2017

PARCCO	TORAYA	AYMARAES	APURIMAC	-73.3029	-14.0895	3/2015	7/2016	11/2017
PISTI	TORAYA	AYMARAES	APURIMAC	-73.2693	-14.082	11/2014	3/2016	7/2017
PUCAPAMPA	TORAYA	AYMARAES	APURIMAC	-73.2878	-14.1025	4/2015	8/2016	11/2017
SARANI	TORAYA	AYMARAES	APURIMAC	-73.2737	-14.0463	8/2014	11/2015	3/2017
TAHUATUNE	TORAYA	AYMARAES	APURIMAC	-73.3046	-14.0533	1/2015	5/2016	8/2017
TANTA	TORAYA	AYMARAES	APURIMAC	-73.2774	-14.0321	8/2014	12/2015	3/2017
TORAYA	TORAYA	AYMARAES	APURIMAC	-73.2946	-14.0531	01/2015	4/2016	7/2017
AMAYAJE / PACCAPATA	YANACA	AYMARAES	APURIMAC	-73.1664	-14.2183	2/2016	6/2017	10/2018
CUYO	YANACA	AYMARAES	APURIMAC	-73.1847	-14.2299	5/2016	9/2017	1/2019
HUARACCOPATA	YANACA	AYMARAES	APURIMAC	-73.1484	-14.2231	3/2016	7/2017	11/2018
INGENIO	YANACA	AYMARAES	APURIMAC	-73.1523	-14.2079	12/2015	4/2017	8/2018
JESUS MARIA	YANACA	AYMARAES	APURIMAC	-73.1375	-14.2051	1/2016	5/2017	9/2018
LLULLUSA	YANACA	AYMARAES	APURIMAC	-73.1401	-14.2433	7/2016	11/2017	4/2019
PACCHATA	YANACA	AYMARAES	APURIMAC	-73.1633	-14.2187	2/2016	6/2017	10/2018
PARANCAY	YANACA	AYMARAES	APURIMAC	-73.1323	-14.2316	5/2016	9/2017	2/2019
PARCO	YANACA	AYMARAES	APURIMAC	-73.1547	-14.2035	12/2015	4/2017	8/2018
PATASAYANA	YANACA	AYMARAES	APURIMAC	-73.1439	-14.2397	6/2016	10/2017	2/2019
PUCULLONE	YANACA	AYMARAES	APURIMAC	-73.1793	-14.2153	3/2016	7/2017	11/2018
PUNTA	YANACA	AYMARAES	APURIMAC	-73.1357	-14.2138	2/2016	7/2017	11/2018
QUILCATA	YANACA	AYMARAES	APURIMAC	-73.1174	-14.2401	7/2016	11/2017	3/2019
QUISUARA	YANACA	AYMARAES	APURIMAC	-73.1594	-14.2051	12/2015	4/2017	8/2018
SARAICA	YANACA	AYMARAES	APURIMAC	-73.1337	-14.2096	2/2016	7/2017	11/2018
TAPURAY	YANACA	AYMARAES	APURIMAC	-73.1508	-14.1952	11/2015	3/2017	7/2018
TUMIRE	YANACA	AYMARAES	APURIMAC	-73.1578	-14.2074	12/2015	4/2017	8/2018
VILQUE	YANACA	AYMARAES	APURIMAC	-73.1563	-14.2149	1/2016	6/2017	10/2018
YANACA	YANACA	AYMARAES	APURIMAC	-73.1602	-14.2259	3/2016	8/2017	12/2018
ACERO	TAMBOBAMBA	COTABAMBAS	APURIMAC	-72.1223	-13.9097	8/2016	12/2017	4/2019

CHACCARO	TAMBOBAMBA	COTABAMBAS	APURIMAC	-72.1359	-13.8486	3/2016	6/2017	9/2018
CHAMAYO	TAMBOBAMBA	COTABAMBAS	APURIMAC	-72.1203	-13.8214	2/2016	5/2017	9/2018
CHUNCHUNA	TAMBOBAMBA	COTABAMBAS	APURIMAC	-72.1149	-13.8635	5/2016	8/2017	12/2018
CHUROC	TAMBOBAMBA	COTABAMBAS	APURIMAC	-72.1901	-13.77	8/2015	11/2016	2/2018
HACIENDA CHAHUARQUI	TAMBOBAMBA	COTABAMBAS	APURIMAC	-72.2163	-13.7486	5/2015	8/2016	11/2017
HACIENDA UCHUBAMBA	TAMBOBAMBA	COTABAMBAS	APURIMAC	-72.1147	-13.8227	3/2016	6/2017	9/2018
MATAC	TAMBOBAMBA	COTABAMBAS	APURIMAC	-72.1323	-13.8256	2/2016	5/2017	8/2018
MISCCA	TAMBOBAMBA	COTABAMBAS	APURIMAC	-72.1522	-13.8285	1/2016	5/2017	8/2018
PAMPARQUI	TAMBOBAMBA	COTABAMBAS	APURIMAC	-72.2327	-13.7469	4/2015	7/2016	9/2017
RACCATI	TAMBOBAMBA	COTABAMBAS	APURIMAC	-72.1634	-13.8092	12/2015	3/2017	6/2018
UCHUPATA	TAMBOBAMBA	COTABAMBAS	APURIMAC	-72.1302	-13.831	2/2016	5/2017	8/2018
YUMACRU	TAMBOBAMBA	COTABAMBAS	APURIMAC	-72.1459	-13.8267	1/2016	5/2017	8/2018
ANÍARQUI	COTABAMBAS	COTABAMBAS	APURIMAC	-72.4148	-13.7033	01/2015	6/2015	8/2016
ACCOMOCCO	COTABAMBAS	COTABAMBAS	APURIMAC	-72.3438	-13.7483	01/2015	11/2015	2/2017
ACCORO	COTABAMBAS	COTABAMBAS	APURIMAC	-72.3618	-13.7514	01/2015	12/2015	2/2017
ANTACATA	COTABAMBAS	COTABAMBAS	APURIMAC	-72.372	-13.7684	01/2015	2/2016	4/2017
CALLAPUNCO	COTABAMBAS	COTABAMBAS	APURIMAC	-72.2813	-13.7249	0/2015	2/2016	5/2017
CCALLA	COTABAMBAS	COTABAMBAS	APURIMAC	-72.3523	-13.7316	01/2015	10/2015	12/2016
CCARANCCA	COTABAMBAS	COTABAMBAS	APURIMAC	-72.3707	-13.7694	01/2015	2/2016	4/2017
CCOCHAPATA	COTABAMBAS	COTABAMBAS	APURIMAC	-72.3524	-13.7246	01/2015	9/2015	11/2016
CHACARAPATA / CHANCARAPATA	COTABAMBAS	COTABAMBAS	APURIMAC	-72.3602	-13.774	01/2015	1/2016	4/2017
CHAMAN	COTABAMBAS	COTABAMBAS	APURIMAC	-72.3628	-13.7661	01/2015	1/2016	3/2017
CHAQUIPUYNO	COTABAMBAS	COTABAMBAS	APURIMAC	-72.3659	-13.7737	01/2015	1/2016	4/2017
CHAUPEC	COTABAMBAS	COTABAMBAS	APURIMAC	-72.4296	-13.7477	01/2015	9/2015	11/2016
CHIHUANAY	COTABAMBAS	COTABAMBAS	APURIMAC	-72.3228	-13.764	01/2015	2/2016	4/2017
CHINCHAYCHUPA	COTABAMBAS	COTABAMBAS	APURIMAC	-72.3633	-13.7644	01/2015	1/2016	3/2017

CHIRANAY	COTABAMBAS	COTABAMBAS	APURIMAC	-72.3703	-13.7301	01/2015	9/2015	12/2016
CHUCCHUNA	COTABAMBAS	COTABAMBAS	APURIMAC	-72.3666	-13.7708	01/2015	1/2016	4/2017
CHUYLLURPAMPA	COTABAMBAS	COTABAMBAS	APURIMAC	-72.4208	-13.7021	01/2015	6/2015	8/2016
COARQUI	COTABAMBAS	COTABAMBAS	APURIMAC	-72.3477	-13.755	01/2015	12/2015	2/2017
COTABAMBAS	COTABAMBAS	COTABAMBAS	APURIMAC	-72.3562	-13.7471	01/2015	11/2015	1/2017
HACIENDA FASAN	COTABAMBAS	COTABAMBAS	APURIMAC	-72.3089	-13.7382	01/2015	12/2015	3/2017
HUACLLI	COTABAMBAS	COTABAMBAS	APURIMAC	-72.3755	-13.7291	01/2015	10/2015	12/2016
HUARCCOY	COTABAMBAS	COTABAMBAS	APURIMAC	-72.3568	-13.7679	01/2015	1/2016	3/2017
INCHISPAMPA	COTABAMBAS	COTABAMBAS	APURIMAC	-72.3424	-13.6848	01/2015	8/2015	10/2016
KAYRANCA	COTABAMBAS	COTABAMBAS	APURIMAC	-72.3445	-13.6862	01/2015	8/2015	10/2016
KECHAROC / QUICHARA	COTABAMBAS	COTABAMBAS	APURIMAC	-72.427	-13.7707	01/2015	11/2015	1/2017
KUTUCTAY	COTABAMBAS	COTABAMBAS	APURIMAC	-72.2639	-13.7013	0/2015	3/2016	5/2017
LITAN PAMPA	COTABAMBAS	COTABAMBAS	APURIMAC	-72.3531	-13.7415	01/2015	11/2015	1/2017
MARANGALLAY	COTABAMBAS	COTABAMBAS	APURIMAC	-72.2739	-13.7119	0/2015	3/2016	5/2017
OCRACA	COTABAMBAS	COTABAMBAS	APURIMAC	-72.293	-13.7404	01/2015	1/2016	4/2017
PAMPAOCCO	COTABAMBAS	COTABAMBAS	APURIMAC	-72.3616	-13.7351	01/2015	10/2015	1/2017
PANTI	COTABAMBAS	COTABAMBAS	APURIMAC	-72.3614	-13.754	01/2015	12/2015	2/2017
POICOTA	COTABAMBAS	COTABAMBAS	APURIMAC	-72.3727	-13.7603	01/2015	1/2016	3/2017
PRUDENCIO	COTABAMBAS	COTABAMBAS	APURIMAC	-72.3596	-13.7653	01/2015	1/2016	3/2017
PUCARA	COTABAMBAS	COTABAMBAS	APURIMAC	-72.357	-13.7711	01/2015	1/2016	3/2017
RALLARQUI	COTABAMBAS	COTABAMBAS	APURIMAC	-72.2326	-13.7058	3/2015	5/2016	8/2017
RANRAPATA / PATA PATA	COTABAMBAS	COTABAMBAS	APURIMAC	-72.3716	-13.712	01/2015	8/2015	10/2016
SAN JUAN	COTABAMBAS	COTABAMBAS	APURIMAC	-72.3392	-13.7669	01/2015	2/2016	4/2017
SIPITECA	COTABAMBAS	COTABAMBAS	APURIMAC	-72.3537	-13.7736	01/2015	1/2016	3/2017
TAMBURO	COTABAMBAS	COTABAMBAS	APURIMAC	-72.3831	-13.7225	01/2015	9/2015	11/2016
UYLLUMPA	COTABAMBAS	COTABAMBAS	APURIMAC	-72.3578	-13.7576	01/2015	12/2015	2/2017

ACPITAN	COYLLURQUI	COTABAMBAS	APURIMAC	-72.4166	-13.8411	3/2015	5/2016	8/2017
ADOBINA	COYLLURQUI	COTABAMBAS	APURIMAC	-72.4422	-13.9198	8/2015	11/2016	2/2018
CCOCCOTQUI	COYLLURQUI	COTABAMBAS	APURIMAC	-72.4583	-13.8184	1/2015	3/2016	6/2017
CCOCCOTQUI / HUACAMIS	COYLLURQUI	COTABAMBAS	APURIMAC	-72.4583	-13.8184	1/2015	3/2016	6/2017
CHAHUAY	COYLLURQUI	COTABAMBAS	APURIMAC	-72.4197	-13.8632	4/2015	7/2016	9/2017
CHAMCHI	COYLLURQUI	COTABAMBAS	APURIMAC	-72.4688	-13.8183	1/2015	4/2016	6/2017
CHOQUECANÍA	COYLLURQUI	COTABAMBAS	APURIMAC	-72.47	-13.8229	1/2015	4/2016	6/2017
CHUYLLULLO	COYLLURQUI	COTABAMBAS	APURIMAC	-72.4319	-13.7838	01/2015	12/2015	2/2017
COLQUEPATA	COYLLURQUI	COTABAMBAS	APURIMAC	-72.4533	-13.8267	2/2015	4/2016	7/2017
COYLLURQUI	COYLLURQUI	COTABAMBAS	APURIMAC	-72.4321	-13.8374	2/2015	4/2016	7/2017
CUICHI	COYLLURQUI	COTABAMBAS	APURIMAC	-72.4543	-13.7847	01/2015	1/2016	3/2017
HACIENDA CCACCAPATA	COYLLURQUI	COTABAMBAS	APURIMAC	-72.4091	-13.8418	3/2015	6/2016	8/2017
HACIENDA NIHUAQUI	COYLLURQUI	COTABAMBAS	APURIMAC	-72.4019	-13.8522	4/2015	7/2016	9/2017
HATUMPAMPA	COYLLURQUI	COTABAMBAS	APURIMAC	-72.4413	-13.7809	01/2015	12/2015	2/2017
HUACAMIS	COYLLURQUI	COTABAMBAS	APURIMAC	-72.4562	-13.8175	1/2015	3/2016	6/2017
HUANCANAPATA	COYLLURQUI	COTABAMBAS	APURIMAC	-72.436	-13.787	01/2015	12/2015	3/2017
HUASURQUE	COYLLURQUI	COTABAMBAS	APURIMAC	-72.4531	-13.8602	4/2015	7/2016	9/2017
JUYUQUI	COYLLURQUI	COTABAMBAS	APURIMAC	-72.4244	-13.851	3/2015	6/2016	9/2017
MANASQUI	COYLLURQUI	COTABAMBAS	APURIMAC	-72.4549	-13.9041	7/2015	10/2016	1/2018
MANZANAYOC	COYLLURQUI	COTABAMBAS	APURIMAC	-72.4433	-13.8131	0/2015	3/2016	5/2017
OSCCOLLO	COYLLURQUI	COTABAMBAS	APURIMAC	-72.4509	-13.9376	9/2015	12/2016	3/2018
OSNOBAMBA	COYLLURQUI	COTABAMBAS	APURIMAC	-72.4493	-13.9312	9/2015	12/2016	3/2018
PACALLAQUE	COYLLURQUI	COTABAMBAS	APURIMAC	-72.4507	-13.9117	8/2015	11/2016	1/2018
PACHARAZO	COYLLURQUI	COTABAMBAS	APURIMAC	-72.4715	-13.8325	2/2015	5/2016	7/2017
PALLPARO	COYLLURQUI	COTABAMBAS	APURIMAC	-72.4456	-13.8208	1/2015	3/2016	6/2017
PFACO	COYLLURQUI	COTABAMBAS	APURIMAC	-72.4572	-13.8255	2/2015	4/2016	7/2017

PUCARUMI	COYLLURQUI	COTABAMBAS	APURIMAC	-72.4471	-13.83	1/2015	4/2016	6/2017
QUILLIRQUI	COYLLURQUI	COTABAMBAS	APURIMAC	-72.4271	-13.8353	2/2015	4/2016	7/2017
QUISHUARPATA	COYLLURQUI	COTABAMBAS	APURIMAC	-72.4267	-13.7841	01/2015	12/2015	2/2017
RACAYPATA	COYLLURQUI	COTABAMBAS	APURIMAC	-72.4572	-13.8047	0/2015	2/2016	5/2017
SECSEKA	COYLLURQUI	COTABAMBAS	APURIMAC	-72.4291	-13.7784	01/2015	11/2015	2/2017
SIJAHUI	COYLLURQUI	COTABAMBAS	APURIMAC	-72.403	-13.8755	6/2015	8/2016	11/2017
TRANCA / TANTA	COYLLURQUI	COTABAMBAS	APURIMAC	-72.4188	-13.8739	5/2015	7/2016	10/2017
VILCARO	COYLLURQUI	COTABAMBAS	APURIMAC	-72.4722	-13.8899	6/2015	9/2016	12/2017
YADQUIRE	COYLLURQUI	COTABAMBAS	APURIMAC	-72.3988	-13.8625	5/2015	7/2016	10/2017
YAHUA YAHUA	COYLLURQUI	COTABAMBAS	APURIMAC	-72.4337	-13.8491	2/2015	5/2016	7/2017
PITIC	MARA	COTABAMBAS	APURIMAC	-72.0754	-14.0509	8/2017	1/2019	6/2020
HUASCARO PATIS	CURPAHUASI	GRAU	APURIMAC	-72.5767	-14.0101	6/2016	10/2017	2/2019
HUAYO CHICO	CURPAHUASI	GRAU	APURIMAC	-72.5769	-14.0051	6/2016	9/2017	1/2019
HUAYO GRANDE	CURPAHUASI	GRAU	APURIMAC	-72.5757	-14.0008	6/2016	9/2017	1/2019
HUINTUNÍA	CURPAHUASI	GRAU	APURIMAC	-72.5734	-13.9839	4/2016	8/2017	11/2018
JATAGOPATA	CURPAHUASI	GRAU	APURIMAC	-72.5704	-13.9943	5/2016	9/2017	12/2018
PUCURUHUAY	CURPAHUASI	GRAU	APURIMAC	-72.5796	-13.9826	4/2016	8/2017	11/2018
TAMBORACCAY	CURPAHUASI	GRAU	APURIMAC	-72.5715	-13.9478	3/2016	7/2017	10/2018
AMARUYOC	GAMARRA	GRAU	APURIMAC	-72.4769	-13.8189	2/2015	4/2016	7/2017
CABRAPATA	GAMARRA	GRAU	APURIMAC	-72.5509	-13.8108	5/2015	8/2016	10/2017
CAJIAMARCA	GAMARRA	GRAU	APURIMAC	-72.5575	-13.7996	4/2015	7/2016	10/2017
CAJIMARCA	GAMARRA	GRAU	APURIMAC	-72.5541	-13.7912	5/2015	7/2016	10/2017
CALLAHUANTA	GAMARRA	GRAU	APURIMAC	-72.4979	-13.8587	5/2015	8/2016	11/2017
CAUTIA	GAMARRA	GRAU	APURIMAC	-72.5043	-13.7909	1/2015	3/2016	6/2017
CCAHUANATI	GAMARRA	GRAU	APURIMAC	-72.5604	-13.8023	5/2015	8/2016	11/2017
CHALLHUAPUQUIO	GAMARRA	GRAU	APURIMAC	-72.4887	-13.7993	0/2015	3/2016	5/2017
CHAMCHIPAMPA	GAMARRA	GRAU	APURIMAC	-72.5881	-13.8119	7/2015	10/2016	1/2018
CHASQUIMOCCO	GAMARRA	GRAU	APURIMAC	-72.5419	-13.9228	11/2015	2/2017	6/2018

COCHAHUACHI	GAMARRA	GRAU	APURIMAC	-72.5875	-13.8406	8/2015	11/2016	2/2018
COLCAHUATE	GAMARRA	GRAU	APURIMAC	-72.4779	-13.7852	01/2015	2/2016	4/2017
COLLAURO	GAMARRA	GRAU	APURIMAC	-72.4861	-13.8784	6/2015	9/2016	12/2017
COTABAMBAS	GAMARRA	GRAU	APURIMAC	-72.543	-13.8048	4/2015	7/2016	10/2017
CRUZPATA	GAMARRA	GRAU	APURIMAC	-72.5828	-13.8071	7/2015	9/2016	12/2017
CUCUPATA	GAMARRA	GRAU	APURIMAC	-72.5342	-13.8003	4/2015	6/2016	9/2017
CULLUSQUI	GAMARRA	GRAU	APURIMAC	-72.5757	-13.8118	7/2015	10/2016	12/2017
HUACORHUATI	GAMARRA	GRAU	APURIMAC	-72.5303	-13.7973	3/2015	5/2016	8/2017
HUALLPARUME	GAMARRA	GRAU	APURIMAC	-72.4943	-13.8772	6/2015	9/2016	12/2017
HUARCORATA	GAMARRA	GRAU	APURIMAC	-72.515	-13.797	2/2015	4/2016	7/2017
HUAYPARA	GAMARRA	GRAU	APURIMAC	-72.5572	-13.7792	5/2015	8/2016	10/2017
HUAYTAHUANÍA	GAMARRA	GRAU	APURIMAC	-72.5719	-13.7949	6/2015	8/2016	11/2017
HUICHAICANÍA	GAMARRA	GRAU	APURIMAC	-72.5416	-13.8042	4/2015	6/2016	9/2017
HUISCAPUQUIO	GAMARRA	GRAU	APURIMAC	-72.5897	-13.8035	7/2015	10/2016	1/2018
ILLICCHIHUA	GAMARRA	GRAU	APURIMAC	-72.4787	-13.8565	4/2015	7/2016	9/2017
JUIVILLE	GAMARRA	GRAU	APURIMAC	-72.5714	-13.8074	6/2015	9/2016	12/2017
KALLATQUI	GAMARRA	GRAU	APURIMAC	-72.5747	-13.8134	6/2015	9/2016	12/2017
LAMBRASPATA	GAMARRA	GRAU	APURIMAC	-72.4877	-13.7897	01/2015	2/2016	4/2017
LLAMARUMI	GAMARRA	GRAU	APURIMAC	-72.4898	-13.8606	5/2015	8/2016	10/2017
LLAMIBAMBA	GAMARRA	GRAU	APURIMAC	-72.525	-13.8047	3/2015	6/2016	8/2017
LLAULLIPATA	GAMARRA	GRAU	APURIMAC	-72.4916	-13.8497	4/2015	6/2016	9/2017
LLAUQUI	GAMARRA	GRAU	APURIMAC	-72.5154	-13.7799	1/2015	4/2016	6/2017
LLICCHIVILCA	GAMARRA	GRAU	APURIMAC	-72.5241	-13.9059	9/2015	12/2016	3/2018
LLOJE	GAMARRA	GRAU	APURIMAC	-72.5286	-13.7883	2/2015	5/2016	8/2017
LUCANA	GAMARRA	GRAU	APURIMAC	-72.5292	-13.7936	3/2015	5/2016	8/2017
MATIBAMBA	GAMARRA	GRAU	APURIMAC	-72.4964	-13.7996	0/2015	3/2016	5/2017
MATTEZAZA / MATACCAZA	GAMARRA	GRAU	APURIMAC	-72.5105	-13.7919	2/2015	4/2016	7/2017

MOLLEBAMBA	GAMARRA	GRAU	APURIMAC	-72.5085	-13.8016	2/2015	4/2016	7/2017
OCRABAMBA	GAMARRA	GRAU	APURIMAC	-72.5295	-13.9285	11/2015	2/2017	5/2018
OLLABAMBA	GAMARRA	GRAU	APURIMAC	-72.4909	-13.7962	0/2015	3/2016	5/2017
PACCAIPATA	GAMARRA	GRAU	APURIMAC	-72.5395	-13.8061	4/2015	6/2016	9/2017
PACCALLAQUI	GAMARRA	GRAU	APURIMAC	-72.4917	-13.7995	0/2015	3/2016	5/2017
PACPIRI	GAMARRA	GRAU	APURIMAC	-72.4937	-13.8698	6/2015	9/2016	11/2017
PALPACACHI	GAMARRA	GRAU	APURIMAC	-72.5083	-13.8721	6/2015	9/2016	12/2017
PAQUERPATA	GAMARRA	GRAU	APURIMAC	-72.5318	-13.8074	3/2015	6/2016	8/2017
PARUBAMBA	GAMARRA	GRAU	APURIMAC	-72.5911	-13.8371	8/2015	11/2016	2/2018
PICHIBAMBA	GAMARRA	GRAU	APURIMAC	-72.5688	-13.8347	7/2015	10/2016	1/2018
PINCULLUNE	GAMARRA	GRAU	APURIMAC	-72.5533	-13.7839	5/2015	7/2016	10/2017
PITUHUANCA	GAMARRA	GRAU	APURIMAC	-72.4961	-13.7856	0/2015	3/2016	5/2017
POMABAMBA	GAMARRA	GRAU	APURIMAC	-72.4725	-13.8483	3/2015	6/2016	9/2017
PUCARACAYPATA	GAMARRA	GRAU	APURIMAC	-72.5362	-13.8154	4/2015	6/2016	9/2017
PUPURQUI	GAMARRA	GRAU	APURIMAC	-72.492	-13.8497	4/2015	7/2016	9/2017
QUENTAS	GAMARRA	GRAU	APURIMAC	-72.4869	-13.8507	4/2015	7/2016	10/2017
SAMPURO	GAMARRA	GRAU	APURIMAC	-72.543	-13.8943	10/2015	2/2017	5/2018
SAN JUAN	GAMARRA	GRAU	APURIMAC	-72.4879	-13.7945	0/2015	3/2016	5/2017
SAPSI	GAMARRA	GRAU	APURIMAC	-72.5547	-13.7917	4/2015	7/2016	10/2017
SARCONTA	GAMARRA	GRAU	APURIMAC	-72.5608	-13.8139	6/2015	8/2016	11/2017
SIUXPAMPA	GAMARRA	GRAU	APURIMAC	-72.5573	-13.8069	5/2015	7/2016	10/2017
TARIBAMBA	GAMARRA	GRAU	APURIMAC	-72.5764	-13.8397	8/2015	10/2016	1/2018
TIMPOSPUQUIO	GAMARRA	GRAU	APURIMAC	-72.561	-13.8339	7/2015	10/2016	12/2017
TRANCAPATA	GAMARRA	GRAU	APURIMAC	-72.566	-13.8035	5/2015	8/2016	11/2017
TRANCAPATA	GAMARRA	GRAU	APURIMAC	-72.566	-13.7989	5/2015	8/2016	11/2017
TTIPIN	GAMARRA	GRAU	APURIMAC	-72.4866	-13.8203	2/2015	4/2016	7/2017
URIHUIRE	GAMARRA	GRAU	APURIMAC	-72.5927	-13.8492	9/2015	12/2016	3/2018
UTAPARU	GAMARRA	GRAU	APURIMAC	-72.4766	-13.7907	01/2015	2/2016	4/2017

AHUAYTANI	HUAYLLATI	GRAU	APURIMAC	-72.5204	-13.9619	2/2016	5/2017	8/2018
CCALLASSOQUE / CALLASOQUE	HUAYLLATI	GRAU	APURIMAC	-72.5333	-13.9465	12/2015	3/2017	7/2018
CCASCCARUMI	HUAYLLATI	GRAU	APURIMAC	-72.4772	-13.918	9/2015	12/2016	3/2018
CHILLEC	HUAYLLATI	GRAU	APURIMAC	-72.461	-13.9449	10/2015	1/2017	4/2018
HUAYLLATI	HUAYLLATI	GRAU	APURIMAC	-72.4845	-13.9283	10/2015	1/2017	4/2018
HUERTA CCATA	HUAYLLATI	GRAU	APURIMAC	-72.4874	-13.9219	9/2015	12/2016	3/2018
KISCAMARCA	HUAYLLATI	GRAU	APURIMAC	-72.4613	-13.9475	10/2015	1/2017	4/2018
PAMPAHUITE	HUAYLLATI	GRAU	APURIMAC	-72.5267	-13.9744	2/2016	5/2017	9/2018
PAYAQUISTE	HUAYLLATI	GRAU	APURIMAC	-72.5547	-13.992	4/2016	8/2017	12/2018
QUISCAPATA	HUAYLLATI	GRAU	APURIMAC	-72.5147	-13.9807	3/2016	6/2017	10/2018
ROCCA	HUAYLLATI	GRAU	APURIMAC	-72.4913	-13.9182	9/2015	12/2016	3/2018
SICLLA	HUAYLLATI	GRAU	APURIMAC	-72.4841	-13.9175	9/2015	12/2016	3/2018
TENERIA	HUAYLLATI	GRAU	APURIMAC	-72.5066	-13.9349	11/2015	2/2017	5/2018
VILCAKI	HUAYLLATI	GRAU	APURIMAC	-72.498	-13.9158	9/2015	12/2016	3/2018
PACCAYURA	PROGRESO	GRAU	APURIMAC	-72.5693	-14.0018	6/2016	9/2017	1/2019
HACIENDA LUCRE	CURASCO	GRAU	APURIMAC	-72.5758	-14.0172	7/2016	11/2017	2/2019
MANCAHUARA	CURASCO	GRAU	APURIMAC	-72.5573	-14.0411	7/2016	11/2017	3/2019
NAHUINQUI	CHINCHAYPUJIO	ANTA	CUSCO	-72.2772	-13.633	01/2015	1/2016	3/2017
ASAJUANA	CHINCHAYPUJIO	ANTA	CUSCO	-72.2514	-13.6506	01/2015	2/2016	4/2017
AYLIAPATA	CHINCHAYPUJIO	ANTA	CUSCO	-72.2188	-13.6484	2/2015	5/2016	7/2017
CAMPANILLAYOC	CHINCHAYPUJIO	ANTA	CUSCO	-72.2876	-13.6457	01/2015	12/2015	2/2017
CAPULIYOC	CHINCHAYPUJIO	ANTA	CUSCO	-72.2113	-13.6616	3/2015	5/2016	8/2017
CARCO	CHINCHAYPUJIO	ANTA	CUSCO	-72.3053	-13.6516	01/2015	10/2015	12/2016
CCALLIS	CHINCHAYPUJIO	ANTA	CUSCO	-72.2383	-13.6063	2/2015	5/2016	7/2017
CCOCHAHUARI	CHINCHAYPUJIO	ANTA	CUSCO	-72.2289	-13.6369	2/2015	4/2016	7/2017
CCOCHAPATA	CHINCHAYPUJIO	ANTA	CUSCO	-72.3518	-13.662	01/2015	7/2015	9/2016
CCONCCAHUAYLLA	CHINCHAYPUJIO	ANTA	CUSCO	-72.2974	-13.6261	01/2015	12/2015	2/2017

CHACACHIMPA	CHINCHAYPUJIO	ANTA	CUSCO	-72.2301	-13.6356	2/2015	4/2016	7/2017
CHACAPAMPA	CHINCHAYPUJIO	ANTA	CUSCO	-72.2222	-13.6221	3/2015	6/2016	8/2017
CHALLASPAMPA	CHINCHAYPUJIO	ANTA	CUSCO	-72.2288	-13.6755	2/2015	5/2016	7/2017
CHILCACCASA	CHINCHAYPUJIO	ANTA	CUSCO	-72.3213	-13.6116	01/2015	11/2015	1/2017
CHILLIHUAY	CHINCHAYPUJIO	ANTA	CUSCO	-72.3011	-13.6488	01/2015	10/2015	1/2017
CHINCHAYPUJIO	CHINCHAYPUJIO	ANTA	CUSCO	-72.233	-13.6295	2/2015	5/2016	7/2017
CHOCHACA	CHINCHAYPUJIO	ANTA	CUSCO	-72.373	-13.6485	01/2015	5/2015	7/2016
CHULLASPAMPA	CHINCHAYPUJIO	ANTA	CUSCO	-72.2314	-13.6251	2/2015	5/2016	7/2017
CHUYPA	CHINCHAYPUJIO	ANTA	CUSCO	-72.3777	-13.6317	01/2015	4/2015	6/2016
COCHAPAMPA	CHINCHAYPUJIO	ANTA	CUSCO	-72.2756	-13.6353	01/2015	1/2016	3/2017
COLLMAY	CHINCHAYPUJIO	ANTA	CUSCO	-72.2649	-13.6173	0/2015	2/2016	5/2017
COLLOCORRAL	CHINCHAYPUJIO	ANTA	CUSCO	-72.3772	-13.6267	01/2015	4/2015	6/2016
CONCAHUAYLLA	CHINCHAYPUJIO	ANTA	CUSCO	-72.2649	-13.6509	01/2015	1/2016	4/2017
CRUZCCERA	CHINCHAYPUJIO	ANTA	CUSCO	-72.2197	-13.6341	2/2015	5/2016	8/2017
CULLPANI	CHINCHAYPUJIO	ANTA	CUSCO	-72.2419	-13.6194	1/2015	4/2016	6/2017
HACIENDA AQUILLAHASI	CHINCHAYPUJIO	ANTA	CUSCO	-72.327	-13.6345	01/2015	9/2015	11/2016
HACIENDA HUANTARO	CHINCHAYPUJIO	ANTA	CUSCO	-72.2373	-13.6752	1/2015	4/2016	6/2017
HACIENDA OSCOLLOPAMPA	CHINCHAYPUJIO	ANTA	CUSCO	-72.3066	-13.6396	01/2015	11/2015	1/2017
HACIENDA SAN JOSE	CHINCHAYPUJIO	ANTA	CUSCO	-72.3502	-13.6817	01/2015	7/2015	9/2016
HAMPPATUYOC	CHINCHAYPUJIO	ANTA	CUSCO	-72.2127	-13.6495	3/2015	5/2016	8/2017
HATUMPAMPA	CHINCHAYPUJIO	ANTA	CUSCO	-72.3187	-13.6199	01/2015	10/2015	12/2016
HUANÍEC	CHINCHAYPUJIO	ANTA	CUSCO	-72.2523	-13.666	0/2015	2/2016	5/2017
HUACCOTO	CHINCHAYPUJIO	ANTA	CUSCO	-72.2251	-13.6499	2/2015	4/2016	7/2017
HUALLPACHACA	CHINCHAYPUJIO	ANTA	CUSCO	-72.3348	-13.6871	01/2015	9/2015	11/2016
HUAMANCHARPA	CHINCHAYPUJIO	ANTA	CUSCO	-72.2225	-13.6886	3/2015	6/2016	8/2017
HUAMBOMAYO	CHINCHAYPUJIO	ANTA	CUSCO	-72.2205	-13.6414	2/2015	5/2016	8/2017

HUANCANCALLA	CHINCHAYPUJIO	ANTA	CUSCO	-72.2231	-13.6708	2/2015	5/2016	8/2017
HUANCARPATA	CHINCHAYPUJIO	ANTA	CUSCO	-72.2288	-13.6805	2/2015	5/2016	7/2017
HUANCARPATA	CHINCHAYPUJIO	ANTA	CUSCO	-72.2691	-13.6351	01/2015	1/2016	4/2017
HUANCHAC	CHINCHAYPUJIO	ANTA	CUSCO	-72.2465	-13.6553	0/2015	3/2016	5/2017
HUANOJRALAY	CHINCHAYPUJIO	ANTA	CUSCO	-72.249	-13.5556	5/2015	8/2016	10/2017
HUATACCULLO	CHINCHAYPUJIO	ANTA	CUSCO	-72.3777	-13.6319	01/2015	4/2015	6/2016
HUAYCCO	CHINCHAYPUJIO	ANTA	CUSCO	-72.2425	-13.6325	1/2015	3/2016	6/2017
HUAYUNCA	CHINCHAYPUJIO	ANTA	CUSCO	-72.2378	-13.6261	1/2015	4/2016	7/2017
IVIMPAMPA	CHINCHAYPUJIO	ANTA	CUSCO	-72.2981	-13.6422	01/2015	11/2015	1/2017
IVIN	CHINCHAYPUJIO	ANTA	CUSCO	-72.3534	-13.6668	01/2015	7/2015	9/2016
JAYLEA	CHINCHAYPUJIO	ANTA	CUSCO	-72.2254	-13.6433	2/2015	4/2016	7/2017
KCANTUPATA	CHINCHAYPUJIO	ANTA	CUSCO	-72.255	-13.5823	3/2015	5/2016	8/2017
LLAULLICANCHA	CHINCHAYPUJIO	ANTA	CUSCO	-72.239	-13.6524	1/2015	3/2016	6/2017
MANCCOQUE	CHINCHAYPUJIO	ANTA	CUSCO	-72.2647	-13.6366	01/2015	2/2016	4/2017
MARANCANCHA	CHINCHAYPUJIO	ANTA	CUSCO	-72.3258	-13.5927	01/2015	12/2015	2/2017
MAUCCAPANTIPATA	CHINCHAYPUJIO	ANTA	CUSCO	-72.3298	-13.5881	01/2015	12/2015	3/2017
MOTOYPAMPA	CHINCHAYPUJIO	ANTA	CUSCO	-72.2697	-13.6194	01/2015	2/2016	4/2017
OSCOLLOCCASA	CHINCHAYPUJIO	ANTA	CUSCO	-72.3108	-13.6394	01/2015	10/2015	12/2016
OSMO	CHINCHAYPUJIO	ANTA	CUSCO	-72.234	-13.5963	2/2015	5/2016	8/2017
PACAS	CHINCHAYPUJIO	ANTA	CUSCO	-72.2101	-13.6859	3/2015	6/2016	9/2017
PACOPATA	CHINCHAYPUJIO	ANTA	CUSCO	-72.2436	-13.5954	2/2015	4/2016	7/2017
PALOMINO	CHINCHAYPUJIO	ANTA	CUSCO	-72.2136	-13.6789	3/2015	6/2016	8/2017
PAMPAERA	CHINCHAYPUJIO	ANTA	CUSCO	-72.2235	-13.6499	2/2015	5/2016	7/2017
PANCAPAMPA	CHINCHAYPUJIO	ANTA	CUSCO	-72.2416	-13.5986	2/2015	5/2016	8/2017
PARCCOTICA	CHINCHAYPUJIO	ANTA	CUSCO	-72.2486	-13.5738	4/2015	6/2016	9/2017
PAUCARCCOTO	CHINCHAYPUJIO	ANTA	CUSCO	-72.2154	-13.6601	3/2015	5/2016	8/2017
PAUCARPATA	CHINCHAYPUJIO	ANTA	CUSCO	-72.2962	-13.6746	01/2015	11/2015	2/2017
PERAHUAYCO	CHINCHAYPUJIO	ANTA	CUSCO	-72.2982	-13.6269	01/2015	12/2015	2/2017

PITUCANCHA	CHINCHAYPUJIO	ANTA	CUSCO	-72.2135	-13.66	3/2015	5/2016	8/2017
PITUHUANCA	CHINCHAYPUJIO	ANTA	CUSCO	-72.2102	-13.6407	3/2015	6/2016	8/2017
POTOTOMA	CHINCHAYPUJIO	ANTA	CUSCO	-72.3149	-13.5839	01/2015	1/2016	3/2017
PUCAHUAYLLA	CHINCHAYPUJIO	ANTA	CUSCO	-72.2719	-13.6229	01/2015	2/2016	4/2017
PUCURAYOC / PUNCURAYAC	CHINCHAYPUJIO	ANTA	CUSCO	-72.2216	-13.6221	3/2015	6/2016	8/2017
PUTUTUNA	CHINCHAYPUJIO	ANTA	CUSCO	-72.3271	-13.5869	01/2015	12/2015	3/2017
QUISHUARPATA	CHINCHAYPUJIO	ANTA	CUSCO	-72.2562	-13.56	4/2015	7/2016	9/2017
RACRARUMI	CHINCHAYPUJIO	ANTA	CUSCO	-72.238	-13.6165	2/2015	5/2016	7/2017
ROCCOTO	CHINCHAYPUJIO	ANTA	CUSCO	-72.3243	-13.6452	01/2015	9/2015	11/2016
ROCOTO	CHINCHAYPUJIO	ANTA	CUSCO	-72.3224	-13.6749	01/2015	9/2015	12/2016
SAMANCA	CHINCHAYPUJIO	ANTA	CUSCO	-72.333	-13.6032	01/2015	11/2015	1/2017
SAYLLAPAMPA	CHINCHAYPUJIO	ANTA	CUSCO	-72.2353	-13.6093	2/2015	5/2016	7/2017
SITOSBAMBA	CHINCHAYPUJIO	ANTA	CUSCO	-72.2541	-13.5795	3/2015	5/2016	8/2017
SUMARO	CHINCHAYPUJIO	ANTA	CUSCO	-72.2736	-13.6321	01/2015	1/2016	4/2017
TAMBORADA	CHINCHAYPUJIO	ANTA	CUSCO	-72.2932	-13.662	01/2015	11/2015	1/2017
TASTAPATA	CHINCHAYPUJIO	ANTA	CUSCO	-72.2116	-13.6613	3/2015	5/2016	8/2017
TTERACCANCHI	CHINCHAYPUJIO	ANTA	CUSCO	-72.2347	-13.6447	1/2015	3/2016	6/2017
TUTAPAYOC	CHINCHAYPUJIO	ANTA	CUSCO	-72.214	-13.6378	3/2015	6/2016	8/2017
YAQUERA	CHINCHAYPUJIO	ANTA	CUSCO	-72.2198	-13.6441	2/2015	5/2016	7/2017
PACCAYERA	CAPACMARCA	CHUMBIVILCAS	CUSCO	-72.0594	-14.0635	8/2017	1/2019	6/2020
PATAHUASI	CAPACMARCA	CHUMBIVILCAS	CUSCO	-72.0395	-14.0807	10/2017	3/2019	8/2020
SAYHUA	CAPACMARCA	CHUMBIVILCAS	CUSCO	-72.066	-14.0701	9/2017	2/2019	7/2020
ANCARPATA	HUANOQUITE	PARURO	CUSCO	-72.1364	-13.7154	10/2015	1/2017	4/2018
ARAVITO	HUANOQUITE	PARURO	CUSCO	-72.1692	-13.7131	7/2015	10/2016	1/2018
CAUCAYA	HUANOQUITE	PARURO	CUSCO	-72.0816	-13.7352	3/2016	6/2017	10/2018
CEDRUNE	HUANOQUITE	PARURO	CUSCO	-72.0255	-13.754	6/2016	10/2017	2/2019
CERRONIOCC	HUANOQUITE	PARURO	CUSCO	-72.0221	-13.7543	7/2016	11/2017	3/2019

COROR	HUANOQUITE	PARURO	CUSCO	-72.107	-13.7537	1/2016	4/2017	8/2018
COYCHA	HUANOQUITE	PARURO	CUSCO	-72.1097	-13.7699	1/2016	4/2017	8/2018
DURAZNIOC	HUANOQUITE	PARURO	CUSCO	-72.0946	-13.7636	2/2016	5/2017	9/2018
HACIENDA ARAYPALLPA	HUANOQUITE	PARURO	CUSCO	-72.1935	-13.7019	5/2015	8/2016	11/2017
HACIENDA CASABLANCA	HUANOQUITE	PARURO	CUSCO	-72.1318	-13.7234	10/2015	2/2017	5/2018
HACIENDA OYLLOMPA	HUANOQUITE	PARURO	CUSCO	-72.1452	-13.7191	9/2015	12/2016	3/2018
HACIENDA PACAS	HUANOQUITE	PARURO	CUSCO	-72.1984	-13.6924	5/2015	8/2016	10/2017
HACIENDA PULPERA	HUANOQUITE	PARURO	CUSCO	-72.1434	-13.7439	10/2015	1/2017	4/2018
HACIENDA QUISHUARES	HUANOQUITE	PARURO	CUSCO	-72.1304	-13.7261	11/2015	2/2017	5/2018
HACIENDA UCHUYPACO	HUANOQUITE	PARURO	CUSCO	-72.1245	-13.7499	12/2015	3/2017	6/2018
HUACAYCALLA	HUANOQUITE	PARURO	CUSCO	-72.0772	-13.7408	3/2016	6/2017	10/2018
HUANCAHUANCA	HUANOQUITE	PARURO	CUSCO	-72.0897	-13.7446	2/2016	5/2017	9/2018
KENKARQUI	HUANOQUITE	PARURO	CUSCO	-72.0894	-13.75	2/2016	6/2017	9/2018
LLAULLIPATA	HUANOQUITE	PARURO	CUSCO	-72.0382	-13.7499	6/2016	10/2017	1/2019
LOS MOLINOS	HUANOQUITE	PARURO	CUSCO	-72.0855	-13.7398	2/2016	6/2017	9/2018
PACO	HUANOQUITE	PARURO	CUSCO	-72.1345	-13.7545	11/2015	2/2017	5/2018
RAPACANCAPAMPA	HUANOQUITE	PARURO	CUSCO	-72.1682	-13.7111	7/2015	10/2016	1/2018
RETAMAYO	HUANOQUITE	PARURO	CUSCO	-72.1325	-13.7448	11/2015	2/2017	5/2018
ROCOTO	HUANOQUITE	PARURO	CUSCO	-72.0823	-13.7616	3/2016	7/2017	10/2018
TUNAMOCO QUEN ÍAPARO	HUANOQUITE	PARURO	CUSCO	-72.1441	-13.7111	9/2015	12/2016	3/2018
UCHUYTUPAYOC	HUANOQUITE	PARURO	CUSCO	-72.0221	-13.7471	7/2016	11/2017	3/2019
VACO GRANDE	HUANOQUITE	PARURO	CUSCO	-72.1371	-13.7578	11/2015	2/2017	5/2018
